# Glial cells in the mammalian olfactory bulb

**DOI:** 10.3389/fncel.2024.1426094

**Published:** 2024-07-16

**Authors:** Dan Zhao, Meigeng Hu, Shaolin Liu

**Affiliations:** Isakson Center for Neurological Disease Research, Department of Physiology and Pharmacology, Department of Biomedical Sciences, University of Georgia College of Veterinary Medicine, Athens, GA, United States

**Keywords:** olfactory bulb, glial cells, function, interaction, neurodegeneration

## Abstract

The mammalian olfactory bulb (OB), an essential part of the olfactory system, plays a critical role in odor detection and neural processing. Historically, research has predominantly focused on the neuronal components of the OB, often overlooking the vital contributions of glial cells. Recent advancements, however, underscore the significant roles that glial cells play within this intricate neural structure. This review discus the diverse functions and dynamics of glial cells in the mammalian OB, mainly focused on astrocytes, microglia, oligodendrocytes, olfactory ensheathing cells, and radial glia cells. Each type of glial contributes uniquely to the OB's functionality, influencing everything from synaptic modulation and neuronal survival to immune defense and axonal guidance. The review features their roles in maintaining neural health, their involvement in neurodegenerative diseases, and their potential in therapeutic applications for neuroregeneration. By providing a comprehensive overview of glial cell types, their mechanisms, and interactions within the OB, this article aims to enhance our understanding of the olfactory system's complexity and the pivotal roles glial cells play in both health and disease.

## Introduction

The sense of smell enriches our daily lives in subtle yet profound ways, such as the comforting scent of home-cooked food or the invigorating aroma of ocean air. This seemingly simple sensory input is actually a highly complex neurological process governed by specialized systems. Mammalian olfaction relies on three main systems (Slotnick and Weiler, 1990): the main olfactory system (Keverne, 1999), the accessory olfactory (vomeronasal) system, and the septal organ (Baum and Cherry, 2015). The main olfactory system is the sole functional olfactory mechanism in humans (Cherry and Baum, 2020) thus has been the primary research focus. As a pivotal structure of this system and initial site of synaptic processing olfactory signals, the olfactory bulb (OB) receives odorant signals from olfactory sensory neurons (OSNs) in the olfactory epithelium and transmits these signals to various downstream processing centers in the temporal lobe, a key part of the limbic system (Sobel et al., 1998; Krusemark et al., 2013; Hackländer and Bermeitinger, 2017). The OB anatomical and cellular organization has been summarized in multiple excellent reviews (Nagayama et al., 2014; Ennis et al., 2015; Burton, 2017). Briefly, this intricate structure comprises multiple layers: glomerular layer (GL) where OSNs synapse with mitral and tufted cells (MTCs) and interneurons; external plexiform layer (EPL) containing synapses among MTCs and local interneurons as well as granule cells; mitral cell layer (MCL) and internal plexiform layer (IPL) respectively housing mitral cell bodies and tufted cell axonal terminations; and the granule cell layer (GCL) that is populated by GABAergic granule cells and axon terminals from cortical and subcortical neurons (Imamura et al., 2020). While the OB neuronal organization and functions have been extensively studied, it is crucial not to overlook the glial cells, the unsung heroes: which have historically been considered as mere neural “support.” The recent research underscores their profound roles in optimizing functionality of and resilience as well as adaptability within the nervous system. Depending on the distribution region and species, glial cells make up ~33% to 66% of the total brain mass (Azevedo et al., 2009; Herculano-Houzel, 2014). Nevertheless, recent data even refute the erstwhile notion that the ratio of glia to neurons in a mouse brain is < 1:1 ratio (Von Bartheld et al., 2016), revealing a nearly balanced presence in humans. Among glial subtypes, astrocytes are the most abundant accounting for 20% to 40% of all glial cells and are instrumental in diverse functions like neuronal survival, ionic regulation, and synaptic modulation (Verkhratsky and Butt, 2013; Vasile et al., 2017). Microglia constitute ~10%−15% of brain cells, serving as primary immune defenders of the central nervous system (CNS) (Dos Santos et al., 2020). Oligodendrocytes (OLs), making up about 20% of brain cells, serve as axonal insulators and contribute to myelin sheath formation, axonal maintenance, and neuronal sustenance (Valério-Gomes et al., 2018). Olfactory ensheathing cells (OECs), as specialized macroglia exhibiting phagocytic and immunoprotective properties, assist in the regeneration and guidance of OSN axons (Huang et al., 2010). Lastly, radial glia cells (RGCs) as multifunctional progenitors are essential for neural development and tissue organization so that disruption of their functions potentially causes developmental disorders (Kriegstein and Alvarez-Buylla, 2009).

In this review, we comprehensively summarize the current knowledge on the multifaceted roles of glial cells in the main OB by focusing on their origination, migration, distribution, morphology, and molecular markers or signaling pathways. We also emphasize their pivotal functions in regulating synapse formation, function, plasticity, and elimination within the OB, as well as their roles in neurodegenerative diseases. By elucidating these specific aspects, we aim to delineate how glial cells contribute to the formation and function of a healthy nervous system, particularly their impact on neuronal development and activity. These insights could be instrumental in advancing our understanding of brain health, developing early intervention strategies to treat neurodegenerative diseases, paving the way for innovative therapeutic applications for neuroregeneration, and ultimately improving therapeutic outcomes in patients.

## Origination, migration and distribution

### Astrocytes

**Figure 1:**
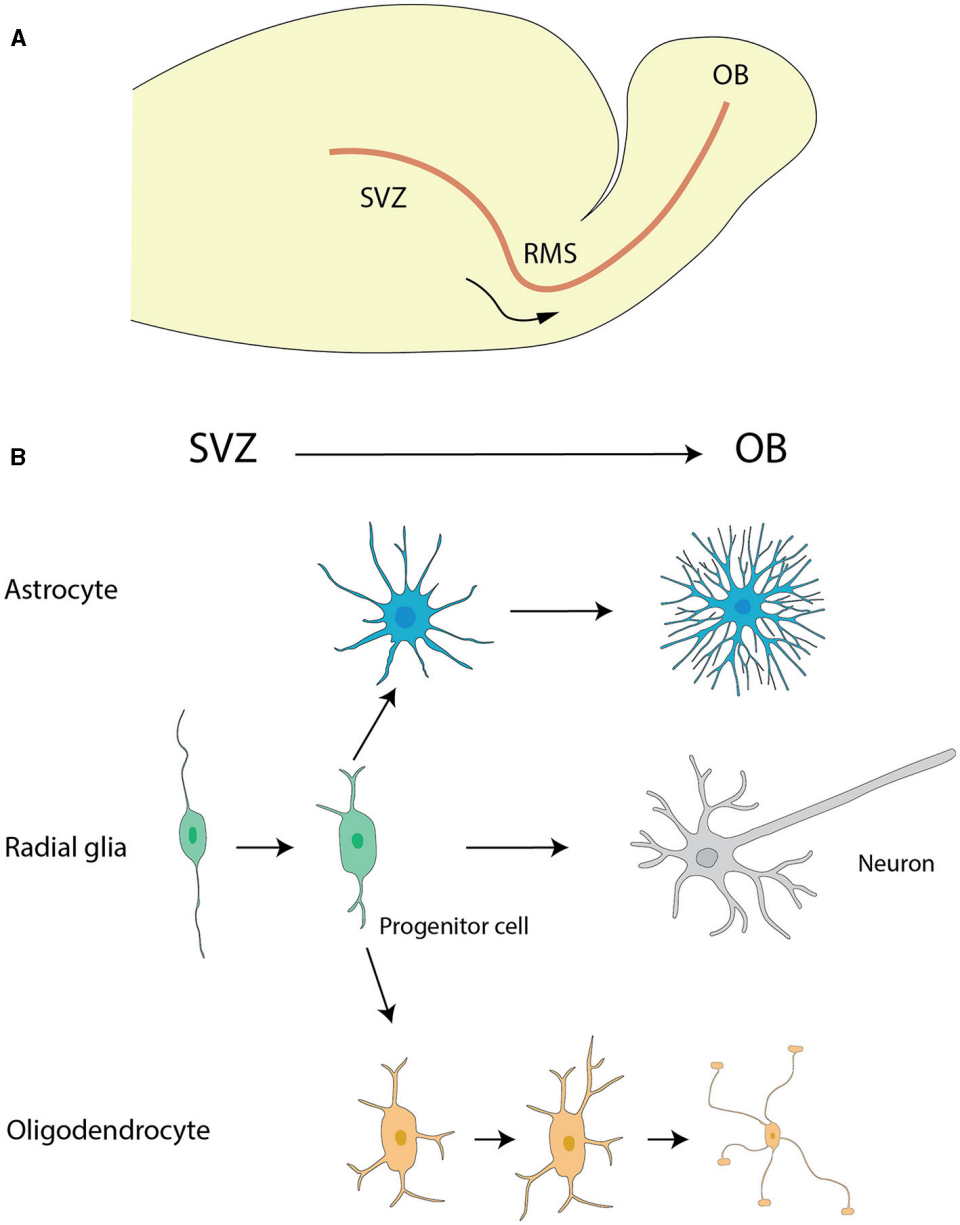
The migration of astrocytes, radial glia and oligodendrocytes. **(A)** The migration pathway of neural progenitor cells from the SVZ to the OB via the RMS. **(B)** Differentiation pathways of radial glial cells and the maturation of astrocytes and oligodendrocytes. Radial glial cells in the SVZ generate neural progenitor cells, which migrate to the OB via the RMS. During their migration, these progenitor cells differentiate into neurons, specifically granule cells and periglomerular cells, within the OB. Additionally, radial glial cells in the SVZ can differentiate into astrocytes and oligodendrocytes. Astrocytes and oligodendrocytes further mature in the SVZ and nearby regions, contributing to the overall functionality and support of the neural environment. The graphic drawings in this figure are based on data from Puche and Shipley (2001) and Belvindrah et al. (2011).

Astrocytes originate from neural progenitor cells and engage in a meticulously structured journey toward the OB during neural development (Zhan et al., 2017). This migration is facilitated through the protrusion of astrocytic extensions that cling to radial glial strands. These strands serve as a structural framework imperative for cellular motility (Ling et al., 1989). The linkage with radial fibers provides not merely guidance for astrocytic trajectory but also contributes to their differentiation process. As they mature, astrocytes assimilate critical characteristics needed to fulfill their duties in preserving cerebral functionality (Figure 1) (Bayraktar et al., 2015).

The rostral migratory stream (RMS) (Figure 1), frequently depicted as a “neuronal highway,” harbors a unique cohort of astrocytes. These varieties of astrocytes serve a pivotal role in orchestrating a combined physical and biochemical corridor that directs the immature neurons toward their terminal—the OB (Peretto et al., 1997; Alonso et al., 2008). This direction is promoted by the emission of chemo-attractive agents such as netrins, slit proteins, semaphorins, ephrins, chemokines, and vascular endothelial growth factor (VEGF) (Alvarez-Buylla and Lim, 2004; Thiriet, 2008; Cayre et al., 2009; Buffo et al., 2010), which act as navigational signals for the neuronal precursors charting their course to the OB.

Upon reaching the OB, astrocytes persist in delivering critical contributions. They are integral to preserving the framework's architectural cohesion and actively engage in the oversight of synaptic activity. Their influence extends through facilitating the integration and fine-tuning of synapses linking incoming neurons to pre-established neural circuits (Zhan et al., 2017). These processes through a range of mechanisms including their interactions with neurons, promote synapse development and maturation, modulate synaptic activity and gliotransmission, regulate ionic balance, and provide energy substrates for neurons (Takano et al., 2006).

The overall migration of astrocytes originating from the subventricular zone (SVZ) of the lateral ventricles to OB (Figure 1A) is a key event for paving the way for their diversified role in neurogenesis and synaptic process. This cell migration highlights the significance of studying astrocyte development and function, which has critical implications for therapeutic approaches targeting neurodevelopmental disorders.

In the OB, astrocytes are identified in all layers but have prominent presence in the glomerular layer (Bailey and Shipley, 1993; Roux et al., 2011; Klein et al., 2020). The glomerular astrocytes exhibit distinctive morphological and functional characteristics that distinguish them from counterparts in other cerebral locales. In layers deep to the glomerular layer, astrocytes take on heteromorphic features with noticeable variations. These celestial-shaped cells has been categorized into three identifiable clusters based on the characteristics of their cytoplasmic extensions emanating from the somas (Bailey and Shipley, 1993; Klein et al., 2020). It is postulated that such alignment divergences among cellular appendages indicate segregated functionalities linked to the OB framework: potentially ranging from spearheading axonal pathfinding during neurodevelopmental intervals to modulating synaptic interconnectivity amid neuronal assemblies (Bailey and Shipley, 1993; Zhang and Barres, 2010; Oberheim et al., 2012).

### Microglia

Microglia, the resident immune cells constituting 5%−12% of all glial cells in the brain, were first identified by Río-Hortega, who intriguingly called them 'third elements' (Lawson et al., 1990). Río-Hortega described and interpreted the presence of microglia in his pioneer effort which detected their existence even at early stages of development. This led him to hypothesize that microglia could be of mesodermal origin found in the pia mater, an innermost layer of meninges lining the CNS (Río-Hortega, 1919; Rio-Hortega, 1932). Following his discovery, scientists went on to undertake considerable scientific studies concerning the exact lineage and derivation of microglial cells, a topic that has continued to generate constant debate among professionals (Rio-Hortega, 1932; Murabe and Sano, 1982). In the beginning, microglia were thought to be of hematopoietic nature due to their morphological and phagocytic similarities to peripheral monocytes, macrophages, and dendritic cells (DCs) (Rio-Hortega, 1939; Murabe and Sano, 1983). Pioneering studies have provided evidence suggesting that microglia derive from embryonic hematopoietic precursors, which begin to colonize in the CNS before the conceptus becomes a fetus (Hutchins et al., 1990; Alliot et al., 1991). This entry into the CNS officially precedes the onset of bone marrow hematopoiesis (Eglitis and Mezey, 1997; Simard and Rivest, 2004). Subsequent research has challenged long held views and instead proposes that microglia are embryonically derived from a cell lineage that gives rise to cells expressing key macrophage-microglial markers, such as CD11b (Mac-1), F4/80 and FcγRIII (Fc-R) (Alliot et al., 1991; Morris et al., 1991). Among others, the origin of this lineage is the early, uncommitted erythromyeloid progenitor (EMP), i.e., the yolk sac (YS) macrophage (Alliot et al., 1999; Ginhoux and Prinz, 2015; Ferrero et al., 2018; Utz et al., 2020). Primitive macrophages originating from the yolk sac (YS) invade the embryo proper by circulating through the cardiovascular campaign and growing through mesenchyme of the brain. Pial surfaces and the developing fourth ventricle are the gateway across, allowing this immigration to the site in the brain rudiment (Cho et al., 2013). Once these original agents of the immune system have breached the barrier, they divide prolifically over a subsequent period of days and maintain themselves in the CNS for the life of the adult. Alternatively, it has been proposed that circulating monocytes are not the only path by which the microglia make their entrance. Instead, sub-ependymal and/or pericyte-based precursors have been joined to the venous system at the end of the lateral ventricles in the brain and even to the vasculature outside of the blood-brain barrier (BBB) (Lewis, 1968; Mori and Leblond, 1969; Barón and Gallego, 1972; Lawson et al., 1992).

Microglia migration to the OB is a characterized multi-step process involving appropriate signaling molecules and cell interactions. Although the modalities are unexplored, many factors have emerged as determinants. The currently most consensual hypothesis is the migration of microglia from the SVZ to the OB through the RMS (Figure 1A), which extend from lateral ventricle and cavum septum pellucidem (Caggiano and Brunjes, 1993; Xavier et al., 2015). Once entering the bulb, microglia go to the deep laminae in particular the GCL close to subependymal area. An alternative hypothesis involves CX3CL1, a critical chemokine that is concurrently proposed to contribute significantly toward the microglial migration to the OB (Ruitenberg et al., 2008). CX3CL1 activates microglia via CX3CR1 receptors, a process leading to subsequent cytoskeletal/membrane changes (Nishiyori et al., 1998; Maciejewski-Lenoir et al., 1999). This is vital for microglial migration to the OB and helps in preserving the brain homeostasis and functions. Additionally, another mechanism has been proposed based on more enthralling discoveries transcending a thorough insight of cells which make up the bone marrow. It should be noted that this analysis entailed the methodical transplant of stem cells expressing green fluorescent protein from the bone marrow to fatally irradiated mice. Astonishingly, these transplanted cells showed an impressive ability to traverse the BBB and invade the brain parenchyma with remarkable accumulations in various regions, including the OB (Simard and Rivest, 2004).

### OLs

The origin and migration of OLs in the OB is a vibrant mosaic of cellular development and intricately interwoven with diverse progenitor cell populations and differentiation cues. OLs in both the OB and subcortical white matter (SCWM) are primarily generated from distinct NG2 progenitor subpopulations residing in the anterior subventricular zone (aSVZ). These include NG2/Er81/Dlx/DC and NG2/Nkx2.2 progenitors, which contribute extensively to neurons and OLs (Aguirre and Gallo, 2004). The OB also harbors a specialized population of oligodendrocyte progenitor cells (OPCs) derived from Nkx2.1-positive progenitors in the medial ganglionic eminence (MGE) and anterior entopeduncular area (AEP) (Kessaris et al., 2006). This adds an additional level of complexity to the source of OB OLs.

Apart from these progenitors, OLs in the OB also originate from proteolipid protein (Plp)-expressing ventricular progenitors within the rostral pallium, forming an independent lineage unaffected by platelet-derived growth factor receptors (PDGFRs) signaling. Intriguingly, the generation of these cells relies on sonic hedgehog signaling akin to their spinal counterparts, but with a delayed differentiation of 4–5 days in the telencephalon, indicating an inherent oligodendrogenic capacity in the OB influenced by heterotopic heterochronies during transplantation (Spassky et al., 2001).

Stem cell-like multipotential precursors in the SVZ and its rostral extension (RE), including its distal portion in the OB, are crucial for the generation of OB OLs. These cells differ from migratory neuroblasts and exhibit diverse functional properties. For instance, cells from the proximal RE more efficiently give rise to OLs, while cells from the distal RE proliferate more slowly, suggesting a stratification of stem cell populations along the SVZ-RE axis (Gritti et al., 2002). In the adult brain, the ventricular-SVZ (V-SVZ), which is rich in cells expressing epidermal growth factor receptor (EGFR) and fibroblast growth factor receptor (FGFR), serves as an indispensable neurogenic incubator (Galvez-Contreras et al., 2016). This area hosts neural stem cells that continually generate neuroblasts and OL progenitor cells (Figure 1) (Menn et al., 2006).

At a fundamental level, the conversion of human OB neural stem cells (OBNSCs) to OPCs and ultimately into OLs is a process centered around differentiation. This involves the upregulation of OPC/OL markers (CNPase, Galc, NG2, MOG, OLIG1, OLIG2, and MBP) and a simultaneous decline in pluripotency markers (Oct4, Sox2, and Nestin) (Marei et al., 2018). Factors like PDGFs, basic fibroblast growth factor (bFGF), and hepatocyte growth factor guide OPC migration, promoting motility and maintaining bipolar migration along the PDGF gradient. In light of these complexities, elucidating the precise mechanisms dictating OL origination and migration within the OB remains a captivating research frontier.

### OECs

The nomenclature of OECs has evolved with the knowledge advancement in neurosciences. In the late 1800s to early 1900s, these cells were referred to as “olfactory Schwann cells.” By the mid-1900s, this designation had changed to “olfactory nerve ensheathing cells” (ONECs). From there, terminology progressed to “olfactory ensheathing glia” (OEG) and olfactory Schwann cells in the late 1900s to 1990s (Barber and Lindsay, 1982; Wozniak, 1993; Franceschini and Barnett, 1996). Today, the most common term is OECs, a name fitting more of the cells' glial character and ensheathing capacity on the olfactory nerve fibers. Indeed, the progression of nomenclature reflected the proliferation of knowledge regarding these remarkable cells in our neural systems.

OECs originate from a source distinct from other glial cells and emerge either from neural crest (PNS glia) or the neural tube (CNS glia). OECs are delivered to OB by the olfactory placode, a transient tissue that forms the olfactory epithelium and olfactory nerves as the nasal cavity is sculpted during early embryogenesis. OECs travel with these olfactory nerves as they stream toward the OB and ultimately settle in the inner two layers of the nascent structure. During this developmental period, the wave of cells, including OEC progenitors, decants from the base of the invaginating olfactory placode. These cells migrate to invest the telencephalic vesicles and frequently move in intimate apposition to an advancing olfactory nerve. They then participate in the assembly of OB at their destination, another distinction between OECs and other glia (Mendoza et al., 1982; Doucette, 1990).

Proper development, maturation and neuronal regeneration of the olfactory system depend on directional migration of OECs. During development, OEC progenitor cells and developing olfactory sensory axons are generated from the olfactory placode and migrate toward the telencephalic vesicle. The primitive OB does not grow in size once the regions of the telencephalon vesicle have been invaded (called evagination) by the growing nerve fibers from the olfactory lobe; these growing nerve fibers then begin to stripe its outer surface. Collectively, these events eventually lead to the formation of a developing OB, which is a crucial structure for the proper functioning of the olfactory system (Mendoza et al., 1982; Farbman and Squinto, 1985; Doucette, 1989). OEC migration is regulated by different factors like calcium channels, GDNF/GFRa-1/Ret pathway, glycoprotein fibulin-3, and the lysophosphatidic acid (Liu et al., 1995; Buckland and Cunningham, 1998; Yan et al., 2003; Windus et al., 2007, 2010; Vukovic et al., 2009). Another key role in influencing OEC migration in addition to dynamic membrane protrusions is lamellipodial waves. These waves on the cells really help them move, because they enable dynamic modulation of membrane protrusions (Lohr et al., 2005, 2007). The cells can easily crawl by utilizing this type of wave on the actin rich membrane. These findings have important implications for our understanding how OECs are able to stimulate the regeneration of nerves in the affected areas and will provide insights into the development and repair of the olfactory system, a unique brain structure continuing to develop new nerve cells throughout an animal's life. Additionally, they may give us some clues on how we might apply them to neural repair in spinal cord injuries, or other types of traumas or diseases involving the nervous system.

OECs are predominantly distributed in the olfactory nerve layer (ONL), the most outer layer of OB. This layer signifies the point of entry for axons from the OSNs into the bulb. OECs are found interspersed among these axonal bundles within the ONL acting as a sheath to direct their growth into the bulb. Consistently, the distribution of these cells across this layer corresponds with the course of the entering OSN axons (Doucette, 1984, 1990; Au et al., 2002).

Some OECs may be seen abutting onto the adjacent GL comprising a smaller population. This would place these cells at the ONL/GL interface. However, this is rare and at other times the GL is not considered a key location for OECs (Au and Roskams, 2003). Additionally, OECs are usually not found in all other OB layers.

### RGCs

As classified neural stem cells (NSCs), RGCs extend uniformly from the ventricle to the pia in most developing cortical structures. In addition to their major contribution to the formation of the cerebral cortex by acting as progenitor of neurons and neuroglia, RGCs play a role in neuronal migration. They are of particular interest in the context of the RMS (Figure 1). The transformation of RGCs into astrocytes deeply affects the migration of neuroblasts from the SVZ to the OB (Merkle et al., 2004) (Figure 1).

In the developing OB, RGCs have processes reaching most of OB layers radially from the GCL to GL (Puche and Shipley, 2001). Interestingly, a group of RGC-like cells were identified in the adult OB with morphological and immunochemical similarities to radial glia present in the OB during embryonic and early postnatal development. They have large circular somata located in the GCL outside the outer border of the RMS/SVZ of the OB with processes radially extending through the GCL toward and into the MCL (Emsley et al., 2012). The recent concept of the RGCs in the OB is expected to clarify the basic mechanism of the neural development and function.

## Morphology

### Astrocytes

**Figure 2:**
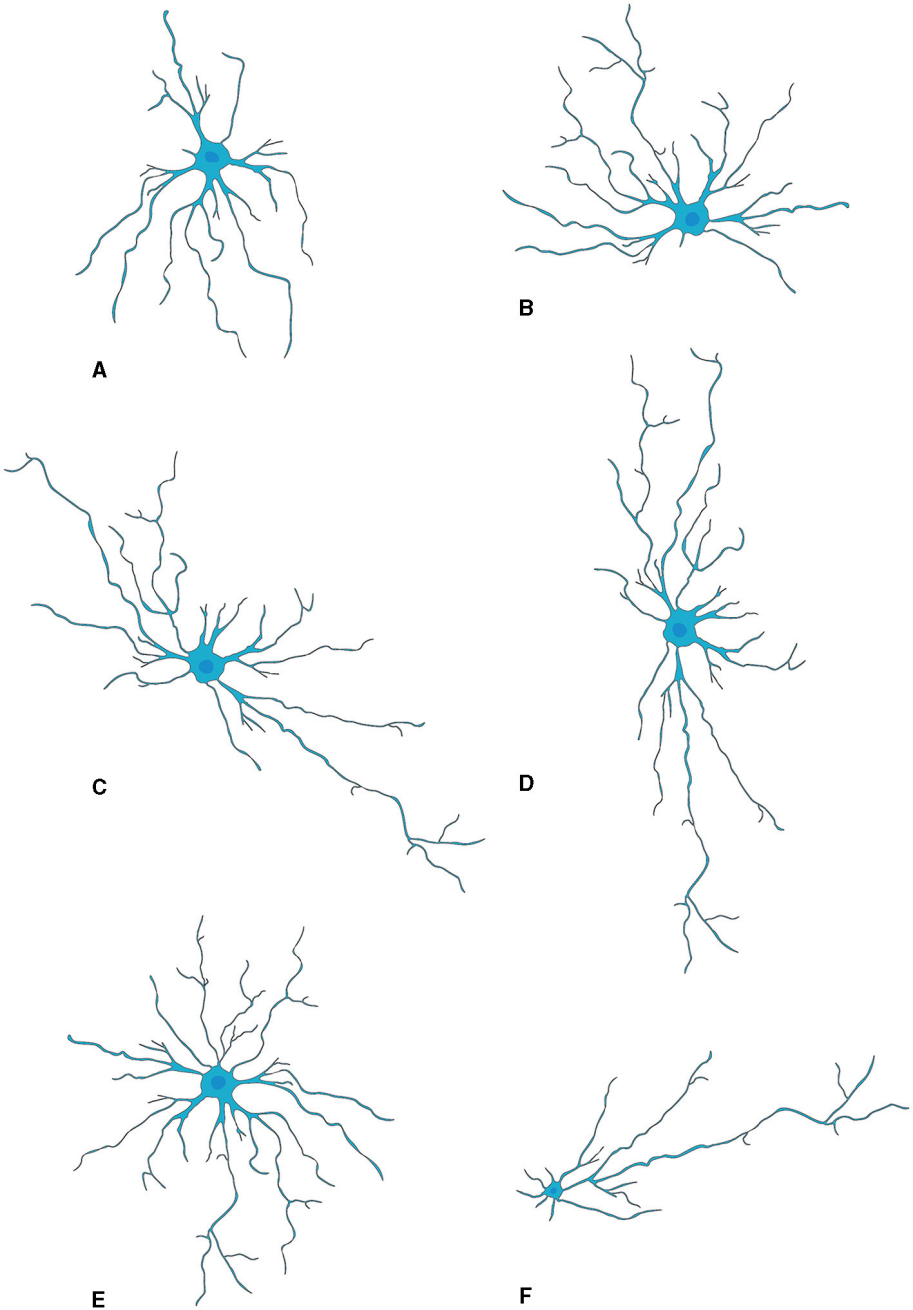
Morphological subtypes of astrocytes in OB. **(A)** Wedge-shape astrocyte. **(B)** Semicircular astrocyte. **(C)** Irregular astrocyte. **(D)** Elongate astrocyte. **(E)** Circular astrocyte. **(F)** Unipolar astrocyte. This figure is conferred from Bailey and Shipley (1993).

The generally star-shaped astrocytes, exhibit notable morphological diversity dependent on their location within the nervous system. In the rat OB, astrocytes were classified into six distinct categories according to their precise locations and their morphological features. Two types of astrocytes found in the glomerular stratum exhibited differentiated shapes, one of which assumed a radial configuration while the other adopted an elongated shape, with extensions pervading multiple or singularly one or two glomeruli respectively. The remaining types of astrocytes were located sequentially in deeper layers EPL, IPL, and GCL, each presenting singular morphologies concomitant with their projected cellular processes (Bailey and Shipley, 1993) (Figure 2).

Recently, Marcel Klein's study delineated three categorically distinct clusters of OB astrocytes (Klein et al., 2020). In the inaugural category, “stellate” astrocytes are typified by a diminutive soma with emerging profuse, finely divided protrusions that intricately wrap around the glomeruli. The next group encompasses “periglomerular” astrocytes, characterized by an ample soma spawning scarcer yet elongated projections that weave into a tight network extending tangentially to the glomerular boundary. The third type involves “protoplasmic” astrocytes which have a formidable soma core flanked by sparser branching tendrils that radiate broadly in assorted directions, integrating into the dense network characteristic of their counterparts. Intriguingly, varied alterations were observed within these defined groups during postnatal neurogenesis: augmentations in length and complexity were observable within both stellate and periglomerular varieties; conversely, protoplasmic forms evidenced negligible modification in morphology.

Moreover, the structural configuration of astrocytes within the OB is subject to modulation as a function of the animal's senescence (Klein et al., 2020). Employing Sholl analytic techniques, the diversity of these glial cells has been discerned in relation to temporal progression (Tavares et al., 2017). A notable transformation, i.e., cellular extensions transition from cephalopod-like formations to more radiating and star-shaped arrangements, is primarily ascribed to alterations within vimentin-positive astrocytic projections. Such findings indicate the presence of persistent structural adaptability in OB resident astrocytes throughout an animal's life course, with potential repercussions on neuronal networks and olfactory function during advanced chronological stages. Nevertheless, elucidation of the underpinning processes precipitating these morphogenic variations and their functional implications necessitates exhaustive exploration.

Appreciating the morphological diversity of astrocytes and their distinct responses to postnatal neurogenesis is critical to understanding their contribution to olfactory processing and other CNS functions. Consequently, continued research in this domain is necessary.

### Microglia

**Figure 3:**
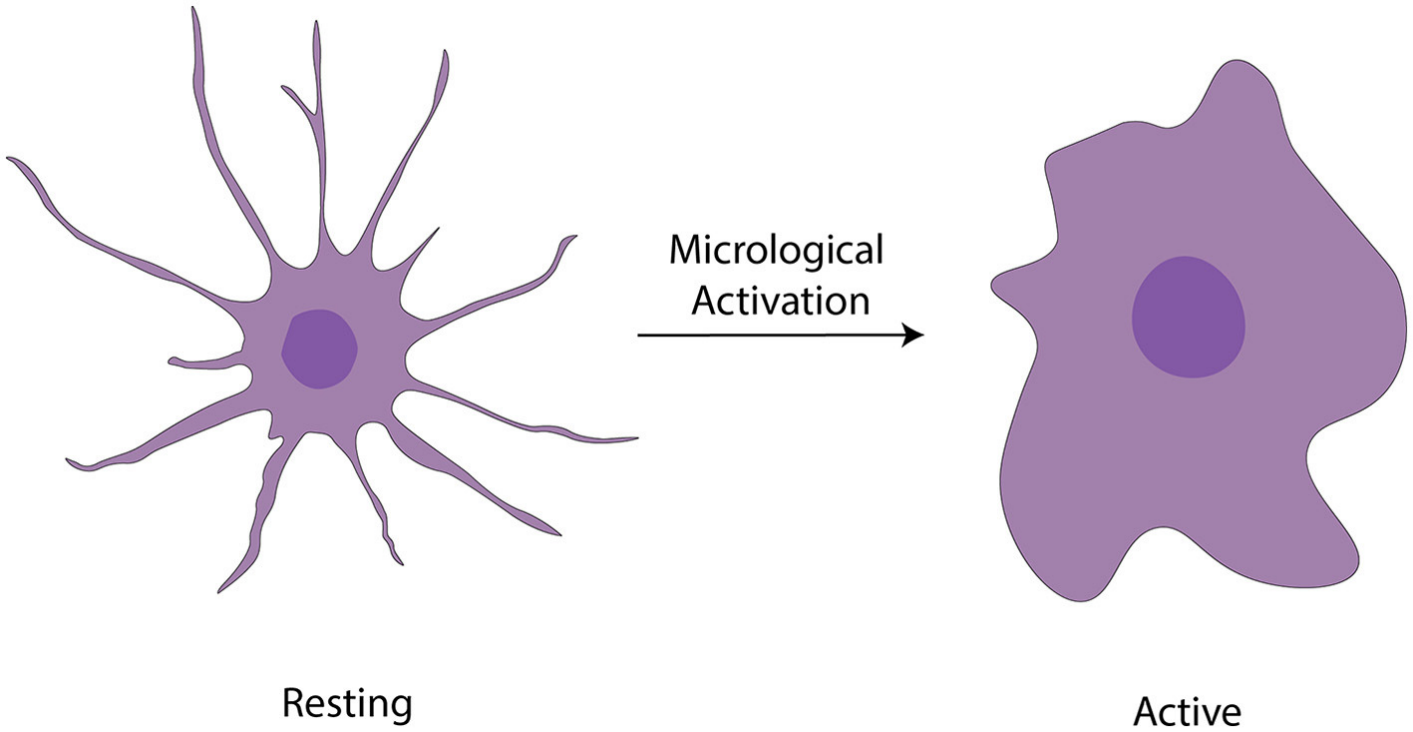
Activation of microglia (Vidal-Itriago et al., 2022).

Microglia are highly abundant in the OB with an average cell density of 127 cells/mm^2^ across all layers, surpassing the density observed in the cortex or neostriatum (Lawson et al., 1990). Two morphological variants of microglia are observed in the OB (Figure 3): a larger, extensively branching, ramified type distributed throughout all layers, and a round-shaped, virtually process-free, amoeboid type principally present in the subependymal zone (Caggiano and Brunjes, 1993). They may represent two different states because microglia with branching have traditionally been considered “resting” while the spherical ones without processes are believed to be “activated” (Rio-Hortega, 1932; Streit et al., 1988, 1999; Stence et al., 2001; Nelson et al., 2002; Rezaie et al., 2002). Researchers used staining techniques involving B4 isolectin derived from *Griffonia simplicifolia* to gain insights into the development of microglia, in the rat OB (Caggiano and Brunjes, 1993). The glomerular layer contained considerable number of ramified cells having cell bodies and processes confined strictly within the boundaries of glomeruli. A fair quantity of amoeboid cells is present in both the olfactory nerve layer and the periglomerular zone (Wu et al., 1997). The ramified microglia with much arborization are scattered throughout the EPL. Also, microglial cell bodies were almost exclusively found in the inner and peripheral layers; microglial processes reach out among the mitral cells. The GCL contains numerous microglial cells, the morphologies of which range from almost ameboid to wholly ramified (Kaplan et al., 1985).

### OLs

**Figure 4:**
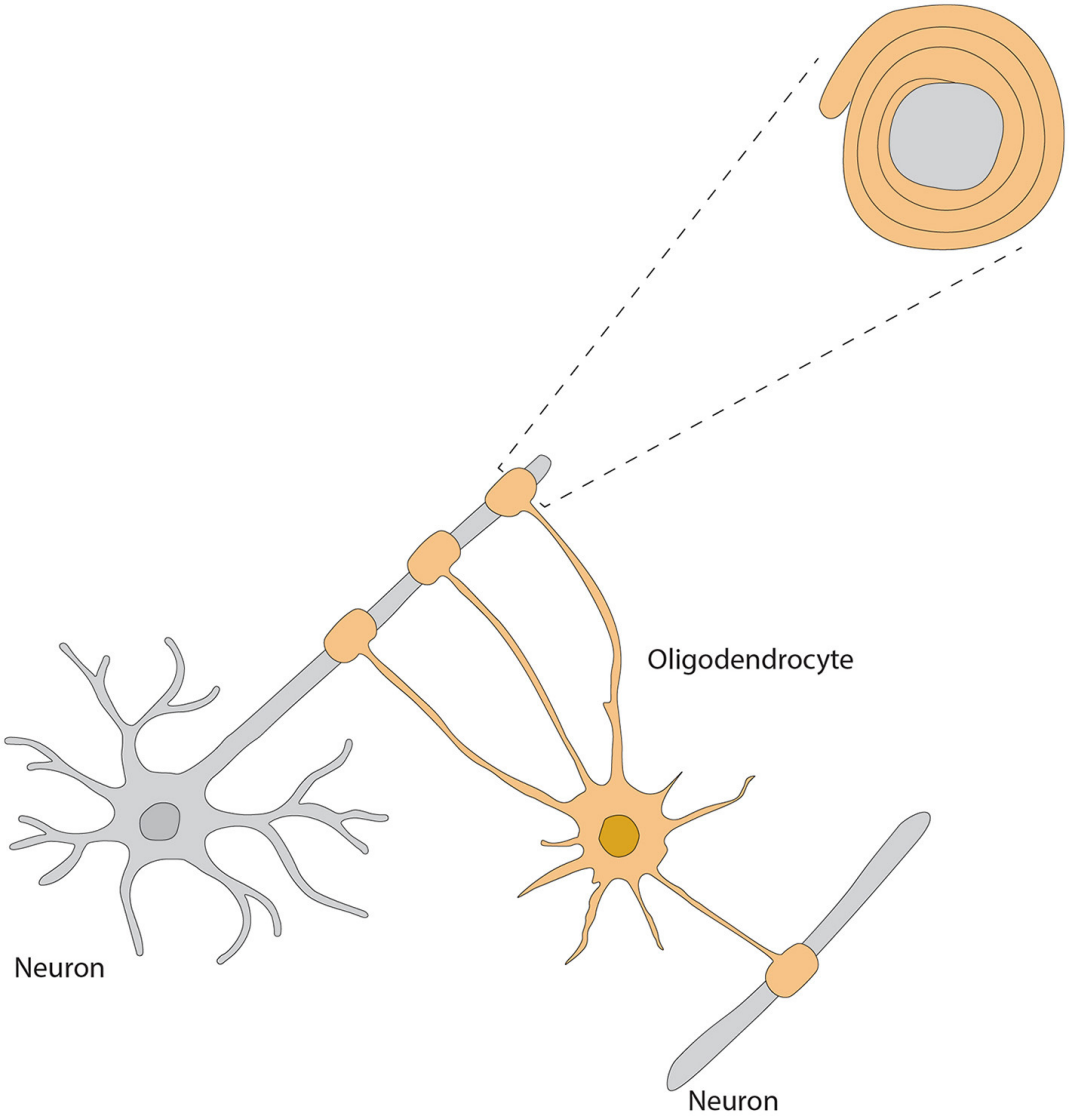
Morphology of oligodendrocyte, referred from Zhang et al. (2021).

As the major myelin-forming glial cells, the OB OLs display differential spatial organization and morphological heterogeneity, thus reflecting their most important roles in modulating neuronal activities, synaptic transmission and axonal conductivity. Two types of OLs, dark- and medium-shaded, can be distinguished by their size and staining characteristics; dark OLs are smaller and constitute approximately 20% of the neuroglial cell population surrounding the glomeruli (Valverde and Lopez-Mascaraque, 1991). Within the GL, they form myelin-like coverings around dendrites and cell bodies within the olfactory glomeruli, potentially modulating synaptic transmission (Zhang et al., 2021) (Figure 4). Such particular distribution highlights the functional importance of OB OLs to rapid processing and conduction of olfactory information.

### OECs

**Figure 5:**
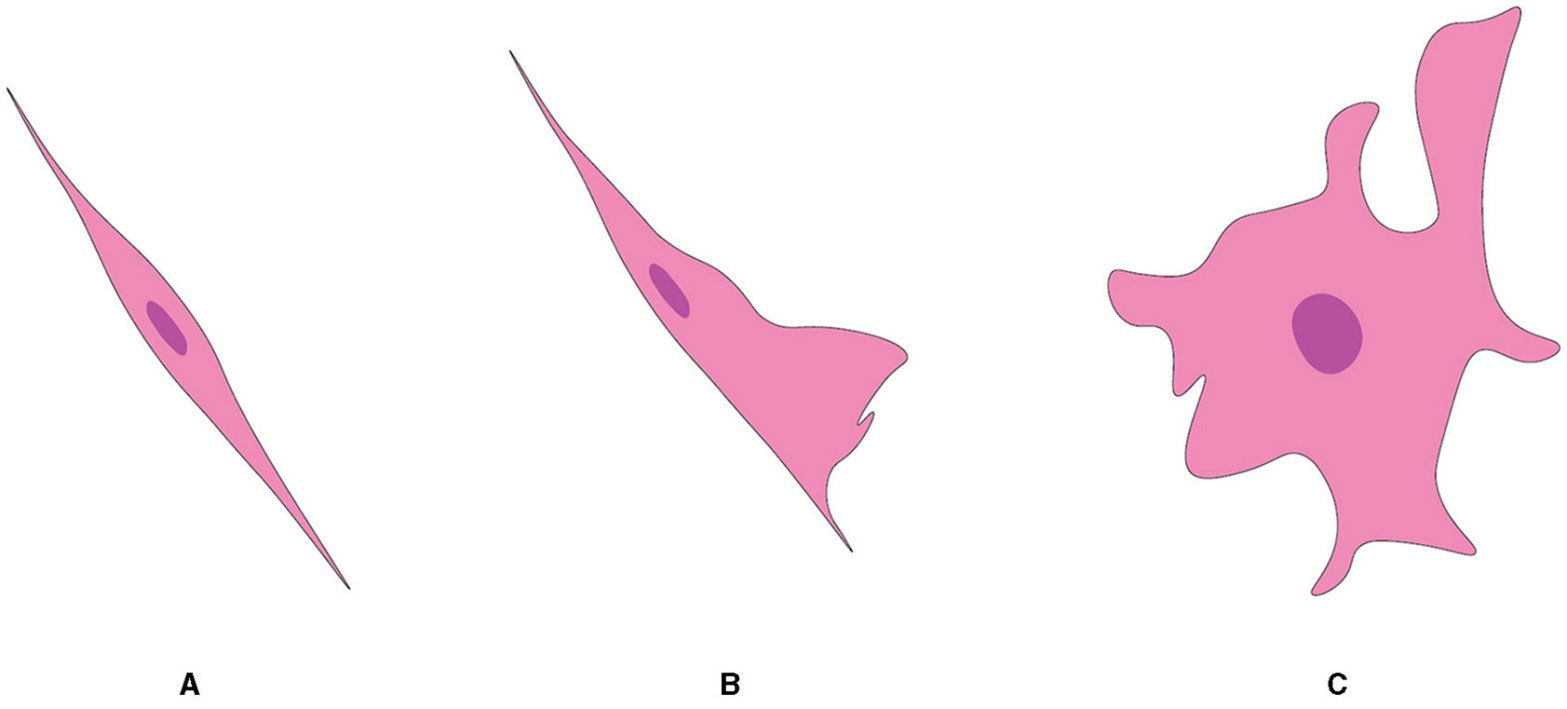
Two subtypes of OEC from OB, referred from Huang et al. (2008). **(A)** Schwann cell-like OEC. **(B)** Type 1 astrocyte-like OEC. **(C)** Type 2 astrocyte-like OEC.

Although they are almost exclusively associated with axons in the nerve fiber layer of the rat OB, OECs exhibit morphological characteristics and plasticity. For example, two morphological subtypes of OECs have been recognized as discrete, characterized mainly by the shape of their structural forms (Figure 5). One subtype has Schwann-like features with processes. In contrast, the other subtype represents the flattened form of astrocytes (Doucette, 1984). Nevertheless, these are not strict classifications but rather points of reference in the continuum of OECs morphological adaptations (Huang et al., 2008).

What distinguishes OECs from other cell types is at the ultrastructural level, which consists of a minimal cytoplasm with a notably electron-dense nucleus. These unique cells typically have a somewhat lobulated nucleus, with the majority of chromatin distributed irregularly around the nuclear envelope. OECs generally contain one or two nucleoli. The electron density of the cytoplasm in an OEC is obviously greater than that in surrounding astrocytes, which do not feature freely scattered intermediate filaments. In terms of overall cellular appearance and as demonstrated best by cells ensheathing olfactory axons in fibers of the nerve layer of the rat OB, OECs look astrocyte-like. However, what renders them unique is that they are able to maintain an embryonic state (Barber and Lindsay, 1982; Doucette, 1984, 1990, 1991; Valverde and Lopez-Mascaraque, 1991).

During their developmental journey, OECs transform in a way that enables some to elongate and extend layered projections. This helps demonstrate the next step in their transformation. At the point where these cells are now mature OECs, they will be seen to surround bundles of small axons. The precursor cells, which are distinguished only as round cells, will eventually turn into mature OECs in the final frame (Valverde et al., 1992).

The ultrastructure of OECs is consistent both *in vivo* and *in vitro*. Mature OECs are attached to the basal lamina in certain areas where they contribute to form external glia limiting membrane within the OB even though they do not have a basal lamina within their plasma membranes, unlike other PNS to CNS transitional zones (Barber and Lindsay, 1982; Doucette, 1984, 1990, 1991). However, they do envelop blood vessels with their processes in a fashion resembling astrocytes.

The essential feature of OECs is their inherent plasticity, which is reflected in their ability to undergo rapid and reversible morphological changes. This dynamic feature provides smooth changes between the two referred subtypes (Li et al., 2018). Thus, the existence of morphological subtypes in OECs is considered as the evidence of their plasticity and versatility. Such plastic alterations are not only spontaneous or arbitrary but also adaptive responses to environmental stimuli, implying a great amount of OEC interaction with the environment. Although this type of flexibility in response to outside factors is a characteristic of these glial cells' dynamic nature and their potential use in neuroregenerative studies, we can uncover other functional aspects of OECs in the olfactory system and elsewhere by investigating their subtypes and dynamic transitions.

### RGCs

RGCs within the OB display an intricate and multifaceted trajectory that contrasts markedly with the uniform extension of their counterparts in other cortical structures (Puche and Shipley, 2001). Specifically, they are characterized by extensive branching and projection patterns that span multiple regions throughout the bulb (Figure 1). Immunohistochemical staining using the RGC cell marker 2 (RC2) antibodies provides an enlightening depiction of the exceedingly branched and elaborate RGCs network in the OB (Hajós and Gallatz, 1987; Anton et al., 1997; Emsley et al., 2012). The stained processes clearly demonstrate RGCs's extensively branched tree-like structure as well as the complex anastomosis formed between RGCs processes. However, the dense packing of the RC2-immunostained processes makes it difficult to unravel the individual branching patterns of these cells, revealing a shortfall in the reliance on immunohistochemistry as the sole investigative method.

To meet this challenge, Puche and Shipley (2001) used the lipophilic membrane dye DiI to label individual cells throughout development. This approach led to two important new revelations. First, individual RGCs can extensively branch within the bulb and able to extend their projections beyond a single glomerulus. Second, while glial glomeruli have prominent features of RGCs during development, stacking of RGCs processes within the glomerular layer appeared to be less prevalent than in other bulb layers. These observations demonstrate that an elaborate and diverse structural configuration of OB RGCs. The complexity of their morphology suggests a significant role in the neural development and functionality within this brain region, underscoring the need for further investigation into their intricate design and operation.

Delving further into the detailed structural morphology, it is important to note that RGCs manifest in two distinct categories, which bear distinctive characteristics and may serve specific functional roles in the development and organization of the OB. One category known as type I RGCs is uniquely defined by a single apical process traveling from the olfactory ventricle to the OB glomerular layer and eventually forming a specialized structure termed “glial glomerulus.” The precise structure of this tuft strongly correlates with the OB glomeruli as well as the burgeoning axons of OSNs. This intricate relationship leads to the speculation that type I RGCs are instrumental in the creation and/or fortification of mammalian OB glomeruli (Puche and Shipley, 2001). In contrast, the second category termed type II RGCs have multiple apical processes that, instead of reaching the glomerular layer, ramify more deeply within the bulb in the EPL. This distinctive feature suggests that type II RGCs play a crucial role in structuring the architecture lamination of the OB. This role may encompass providing a scaffold for the migratory patterns of primary projection neurons, including mitral and tufted cells.

## Molecular markers and signaling

### Astrocytes

Astrocytes are distinguishable from analogous cell types by virtue of specific proteins that they express. They are well known to produce glial fibrillary acidic protein (GFAP), a type of intermediate filament protein essential for cytoarchitectural maintenance and cellular response modulation upon harmful stimuli, making it a reliable astrocytic indicator (Barber and Lindsay, 1982; Doucette, 1984; Bailey and Shipley, 1993). Furthermore, the presence of S100 Calcium-Binding Protein B (S100β) within the cytoplasm acts as another definitive signature of astrocytes. This protein orchestrates diverse intracellular functions and stands as an indicative marker in contexts such as neurotrauma and neuroinflammation (Pixley, 1992; Henriksson and Tjälve, 2000; Haratizadeh et al., 2023). The employment of GFAP staining is predominantly directed toward discerning the nuanced morphological attributes of astrocytes; whereas S100 immunostaining—regularly appearing in tandem with GFAP signals—distinctly delineates the central region or soma of these glial cells.

Aldehyde dehydrogenase 1 family member L1 (ALDH1L1), a protein vital to the body's folate metabolism, serves as another reliable indicator for astrocyte identification (Otsu et al., 2015; Ung et al., 2020, 2021). Furthermore, the protein GLT-1 commonly accompanying ALDH1L1 and facilitating glutamate neurotransmission, is also considered indicative of astrocytic presence. Within the context of the OB, astrocytes often manifest connexin 30—a protein integral to cellular communication through gap junctions, rendering it an efficacious marker for their locative identification in this region (Otsu et al., 2015) even though some juxtaglomerular neurons also express this gap junction protein.

Immunoreactivity of aquaporin-4 (AQP4), a water channel protein vital for maintaining water homeostasis in the CNS (Saadoun and Papadopoulos, 2010) is present in the astrocytic perivascular end-feet around blood vessels in all OB layers. But its intense expression is in astrocytic processes and end-feet surrounding capillaries in the glomerular layer, indicating maintaining water homeostasis is an essential process within the OB for odor signal transduction (Saadoun and Papadopoulos, 2010) (Table 1).

**Table 1:** Overview of glia cells maker proteins.

**Marker**	**Full name**	**References**
**Astrocyte markers**		
GFAP	Glial fibrillary acidic protein	(Barber and Lindsay, 1982; Doucette, 1984; Bailey and Shipley, 1993)
S100β	S100 Calcium-Binding Protein B	(Pixley, 1992; Henriksson and Tjälve, 2000; Haratizadeh et al., 2023)
ALDH1L1	Aldehyde dehydrogenase 1 family member L1	(Otsu et al., 2015; Ung et al., 2020, 2021)
GLT-1	Glutamate transporter 1	(Otsu et al., 2015)
AQP4	Aquaporin-4	(Saadoun and Papadopoulos, 2010)
**Microglia markers**		
TMEM119	Transmembrane protein 119	(Imai et al., 1996; Ito et al., 2001)
CD68	Cluster of differentiation 68	(Hendrickx et al., 2017)
P2Y12	P2Y purinoceptor 12	(Butovsky et al., 2014)
CX3CR1	CX3C motif chemokine receptor 1	(Saederup et al., 2010)
CD40	Cluster of differentiation 40	(Benveniste et al., 2004)
TREM2	Myeloid cells 2	(Ulland and Colonna, 2018)
**Oligodendrocyte markers**		
MBP	Myelin basic protein	(Shinar and McMorris, 1995; Marques et al., 2016)
PLP	Proteolipid protein	(Shinar and McMorris, 1995; Marques et al., 2016)
MAG	Myelin-associated glycoprotein	(Shinar and McMorris, 1995; Marques et al., 2016)
MOG	Myelin oligodendrocyte glycoprotein	(Shinar and McMorris, 1995; Marques et al., 2016)
**Olfactory ensheathing cell markers**		
Rat 401	Anti-Rat Nestin Antibody, Clone Rat401	(Wang et al., 2007; Hwang et al., 2008)
GFAP	Glial fibrillary acidic protein	(Wang et al., 2007; Hwang et al., 2008)
GAP-43	Anti-growth-associated protein 43	(Franceschini and Barnett, 1996)
Gal-C	Anti-galactocerebroside	(Franceschini and Barnett, 1996)
HNK-1	Human Natural Killer 1	(Bock et al., 2007)
1E8	1E8 antibody	(Norgren Jr et al., 1992)
**Radial glia cell markers**		
GFAP	Glial fibrillary acidic protein	(Amaya et al., 2015)
BLBP	Anti-brain lipid-binding protein	(Amaya et al., 2015)
GS	Glutamine synthetase	(Docampo-Seara et al., 2022)
RBA1	Rat brain astrocyte-1	(Pixley and de Vellis, 1984)

Identification of molecular biomarkers for astrocytes provide essential insights into their functionality, reactivity, and adaptability within the OB. These cell membrane-embedded molecules could serve not only as specific markers for determining astrocytic functions at the behavioral or physiological levels by selective manipulation or measures of astrocyte activities with chemogenetic approaches or calcium imaging but also as targets for modulation in diseases affecting the olfactory system. Furthermore, considering the diversity and complexity of astrocyte functions, future research should aim to uncover the precise mechanisms through which these cells influence OB functional dynamics and how these mechanisms may be affected or modulated in pathological conditions.

### Microglia

Microglia have a wide array of molecular footprints, which help us understand the complexity of their functions and interplay of these cells within the neural environment. Iba1, a calcium binding protein closely associated with the activation of microglia and macrophages along with the transmembrane protein 119 (TMEM119) (Imai et al., 1996; Ito et al., 2001), acts as a marker to differentiate between resident microglia and infiltrating macrophages (Bennett et al., 2016). Cluster of differentiation 68 (CD68) (Hendrickx et al., 2017), a glycoprotein extremely upregulated in activated microglia, is used as an indicative marker for the initiation of neuroinflammatory conditions, whilst P2Y12, a purinergic receptor exclusively expressed by surveillant or “resting” microglia, illustrates vital insights to the microglial nature under quiescence (Butovsky et al., 2014). The receptors for fractalkine (CX3CR1) and antigen presenting molecule (CD40) expressed on microglia not only further enhance the complexity of this molecular architecture (Benveniste et al., 2004; Saederup et al., 2010) but also, due to their responses to the ligands on neurons, greatly complicate the communication between the two as well as the immune response potentiation (Ponomarev et al., 2006). Additionally, the triggering receptor expressed on myeloid cells 2 (TREM2) is a modulator of microglial activity and linked to neurodegenerative disorders, particularly AD (Ulland and Colonna, 2018) (Table 1). Collectively, these molecular markers completely underline certain roles microglia play in the CNS and initiate a huge amount of functional immune responses. A greater understanding of these biologic processes will assist in developing therapeutic strategies that address neurodegenerative and neuroinflammatory disorders.

### OLs

An OL is identified by its expression of several key proteins that are essential for maintaining the integrity of compacted myelin sheaths. Myelin basic protein (MBP) comprises a family of proteins with various isoforms that play a crucial role in maintaining the structural integrity of the myelin sheaths. Proteolipid protein (PLP), the major component of myelin, is acylated and contributes to the stability of the myelin membrane. Myelin-associated glycoprotein (MAG) and myelin oligodendrocyte glycoprotein (MOG) are glycoproteins found in myelin, with MOG often being a target for autoimmune responses in demyelinating diseases. Collectively, these proteins maintain the integrity of compacted myelin sheaths (Shinar and McMorris, 1995; Marques et al., 2016) (Table 1).

As crucial components of the CNS, OLs are key to neuron survival and functionality through the secretion of a variety of molecules. Their secretory spectrum includes growth factors such as brain-derived neurotrophic factor (BDNF), which supports neuron survival and differentiation; cytokines including interleukin-1β (IL-1β), tumor necrosis factor-alpha (TNF-α), and transforming growth factor-beta (TGF-β), which play crucial roles in cell signaling; and structural proteins like laminin and fibronectin, contributing to the structural integrity of the nervous system (Muñoz-Fernández and Fresno, 1998). Additionally, OLs release glutathione, an antioxidant that helps protect cells from oxidative stress, the neurotransmitter glutamate essential for neuronal communication, adenosine triphosphate (ATP) as an energy source for cellular processes, and Nogo-A, a protein known for its role as a neurite outgrowth inhibitor, influencing the growth and connectivity of neurons (Anjum et al., 2020). Their functionality is heavily dependent on numerous external factors. The proliferation of OL precursor cells (OPCs) is driven by astrocyte-derived growth factors, with platelet-derived growth factor (PDGF) acting as a potent mitogen and stimulating their proliferation, while fibroblast growth factors (FGFs), a family of signaling proteins with diverse roles in development, tissue repair, and angiogenesis, particularly FGF2, significantly promote the proliferation of neonatal oligodendrocyte progenitors and influence oligodendrocyte development and myelination (Hinks and Franklin, 1999). Insulin-like growth factor-1 (IGF-1) is a critical facilitator of these cells' maturation and the remyelination process. Their survival, proliferation, differentiation, and myelinating activity depend on a host of factors including: neuregulins, ciliary neurotrophic factor (CNTF), leukemia inhibitory factor (LIF), thyroid hormones, and neurotrophins (Domingues et al., 2016). During CNS development, PDGF, Netrin-1, and Shh are representative of long-range extracellular signals that guide OPCs' extensive migratory sojourns (Spassky et al., 2001; Zhang et al., 2004; Bin et al., 2013). Their motility and resultant migratory speed and direction are influenced by neurotransmitters, chemokines, and extracellular matrix proteins. With respect to OPC positioning within the CNS, their gestural interactions with neurons, astrocytes, and extracellular matrix proteins are pivotal. Notably, transcription factors Olig-1 and Olig-2 have pivotal roles in this context. Olig-2 is critical for OPC specification and proliferation, but Olig-1′s presence is required for the transition from OPC to matured OLs (Takebayashi et al., 2000). Dysregulation or mutations in these factors can lead to various neurological disorders, including multiple sclerosis and leukodystrophies, characterized by myelin formation and maintenance defects. CARNS1, a protein participating in the synthesis of histidine-containing dipeptides (HCDs), is identified in OLs of human white matter. Double-labeling of CARNS1 and OLIG2, an OL lineage marker, confirms the presence of CARNS1 within these cells (Van der Stede et al., 2023). OLs secrete extracellular vesicles (EVs) containing molecules such as unprocessed proteolipid protein (PLP) and DM20, two abundant myelin proteins produced through alternative splicing from the PLP gene (Krämer-Albers et al., 2007). Additionally, these EVs can influence neurons by supporting their metabolism and improving their viability under stress conditions, including oxidative stress, starvation, and oxygen and glucose deprivation (OGD) (Frühbeis et al., 2013; Fröhlich et al., 2014).

### OECs

OECs in embryos, neonates, and adults contain nestin (Rat 401) and GFAP (Wang et al., 2007; Hwang et al., 2008). The recent development of two monoclonal antibodies that label the OEC cytoskeleton suggests that they may target a protein within the OEC cytoskeleton that has not yet been identified. Researchers have used antibodies such as anti-growth-associated protein 43 (GAP-43), anti-galactocerebroside (Gal-C), HNK-1 (Human Natural Killer), and 1E8 to study OEC properties (Verhaagen et al., 1989; Franceschini and Barnett, 1996; Bock et al., 2007). The 1E8 antibody, which detects a subset of migratory neural crest cells, Schwann cells, and OECs, has been utilized to further characterize OECs (Norgren Jr et al., 1992) (Table 1). Despite the continuously growing list of membrane proteins known in OECs, identification of new specific markers and molecules expressed in OECs during various developmental stages will help us depict their unique contribution to the neural development of the olfactory system.

Throughout the growth of OECs, there is considerable regulation in expression of the low-affinity nerve growth factor receptor (L-NGFR). Studies have found that axotomy boosts L-NGFR expression in adult olfactory nerves (Barnett et al., 1993, 2000; Turner and Perez-Polo, 1993; Gong et al., 1994; Franceschini and Barnett, 1996; Barnett and Roskams, 2008). OECs consistently express membrane molecules, such as neural cell adhesion molecule L1, laminin, polysialylated neuronal cell adhesion molecule (PSA-N-CAM), and adult neural cell adhesion molecule (N-CAM), which are implicated in cell adhesion and axonal growth across all developmental stages (Miragall et al., 1988; Au et al., 2002; Ramer et al., 2004; Kumar et al., 2005; Planagumà et al., 2011; Witheford et al., 2013; Lazzari et al., 2014; Li et al., 2018). Among them, N-CAM and L1 are specifically localized in the axon-associated segment of the glial membrane, while PSA-N-CAM is only detectable in OECs that constitute the glia limitans, particularly in the membrane region without basal lamina contact (Miragall et al., 1988). Type IV collagen and fibronectin are associated with the OEC membrane region that interacts with the basal lamina (Au and Roskams, 2003; Lakatos et al., 2003; Teng et al., 2008). In developing and maturing OECs, vimentin is the main component of intermediate filaments (Oprych et al., 2017; Pellitteri et al., 2017).

### RGCs

RGCs in the OB display particular glial markers, primarily GFAP, anti-brain lipid-binding protein (BLBP), and glutamine synthetase (GS) (Amaya et al., 2015; Docampo-Seara et al., 2022). These markers have been observed in cells with a radial morphology lining the OB ventricle during embryonic development, and their expression continues into the early juvenile stage, notably in ependymal cells or tanycytes.

The GFAP marker which is most commonly employed for the identification of astrocytes, is expressed by RGCs in the developing brain including the OB (Ventura and Goldman, 2007; Zhu et al., 2018). The presence of GFAP in RGCs assists in discriminating and characterizing them. This marker is typified from cells with a radial layout lining the OB ventricle throughout embryonic development and broadens its expression into tanycytes during early juvenile stages.

Similarly, the marker BLBP, known to be specific to RGCs, is also expressed in the RGCs of the OB's ventricular zone. BLBP expression supports the identification and further analysis of these cells (Theofilas et al., 2017). GS, an enzyme integral to glutamate metabolism, is another marker expressed by RGCs in the OB (Guerrero-Cázares et al., 2011). The GS expression in RGCs contributes to the identification and further characterization of these cells. Echoing the pattern observed with GFAP, both BLBP and GS are expressed in radially oriented cells in the OB ventricle during embryonic stages, this expression is carried forward into tanycytes in the early juvenile stage.

Furthermore, the intermediate filament protein Vimentin is remarkably expressed in RGCs (Pixley et al., 1984). Evidence of this expression is provided by the immunoreactivity of cells labeled with the rat brain astrocyte-1 (RBA1) antibodies, which reportedly identify vimentin (Table 1). The distinct patterns of RBA1-positive cells, including RGCs, spark intriguing theories and questions about the function of vimentin in astrocytes and its possible regulation during recovery from injury or during developmental processes (Pixley and de Vellis, 1984).

Upon differentiation into mature astrocytes, RGCs display a fascinating protein expression dynamic. Typically occurring during the second and third postnatal weeks, it is characterized by a large decline in vimentin expression. Simultaneously, an increase in the intermediate filament protein, GFAP, is noted. Thus, RGCs display a way of altering the appearance of vimentin while they transition into mature astrocytes. This shift emphasizes that proteins in cellular development interact in complicated ways.

## Functions

### Astrocytes

Astrocytes are strategically positioned to spans across crucial cerebral regions encompassing the SVZ and RMS with extension into the OB. This endows them not only with a pivotal role in the intricate process of neurogenesis, the framework, movement, and assimilation of nascent neurons into the OB (Doetsch et al., 1999) but also with their multifaceted functions across varied neurological circumstances (Lim and Alvarez-Buylla, 1999).

#### Structural and metabolic support

**Figure 6:**
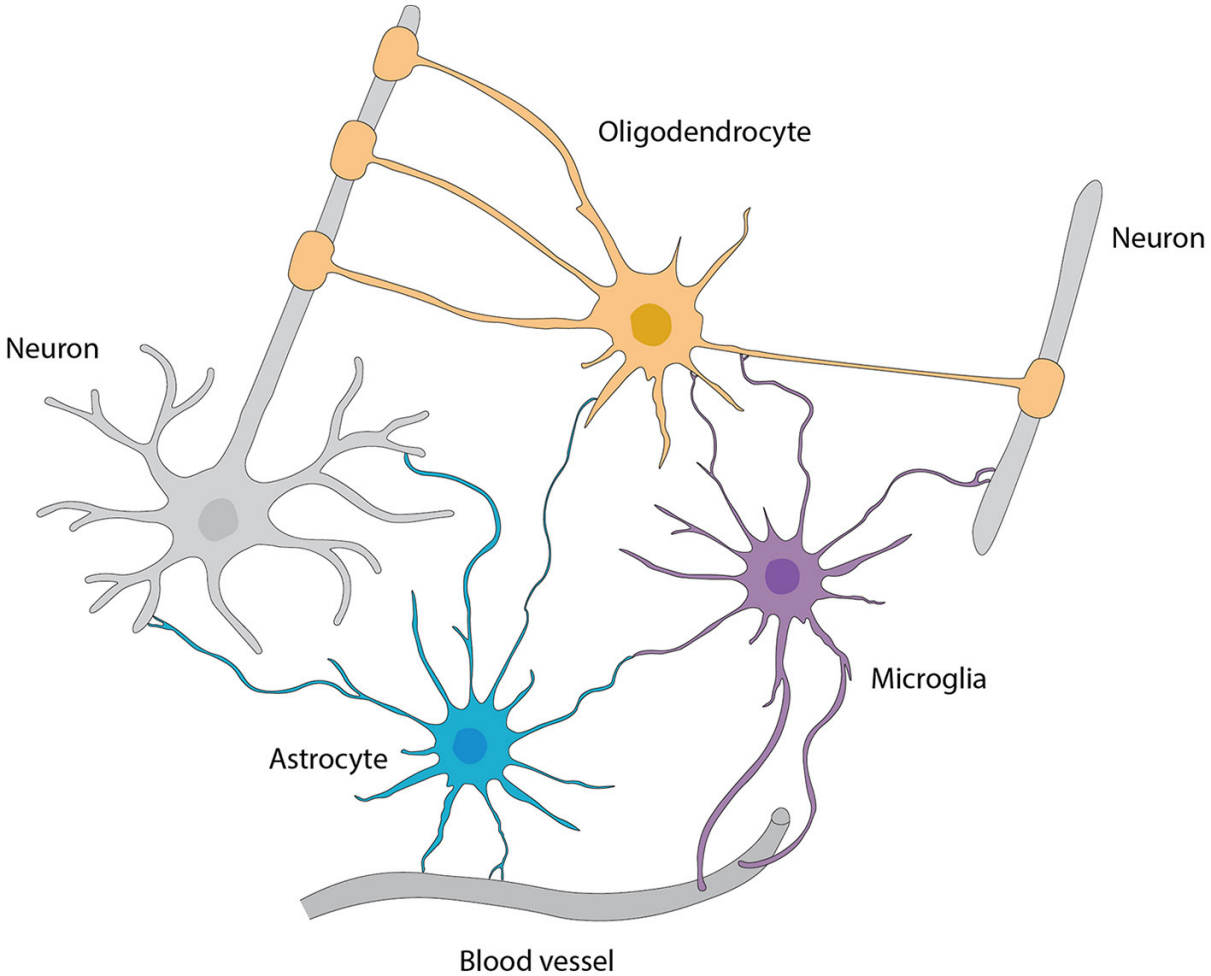
Interactions among various types of glial cells, neurons, and blood vessels, referred from Allen and Lyons (2018).

Astrocytes are the main type of glial cells making up to ~50% of the mammalian brain. At the ultrastructural level, astrocytes' ultrathin processes as “tripartite synapse” architectural elements enwrap the synaptic elements of neurons (Araque et al., 1999). Tripartite synapses in the CNS are estimated to be from 30% to 60% in the neocortex and reaching up to 90% in hippocampus by studies with EM three-dimensional reconstruction. These numbers are very likely underappreciated given the ultra-thinness of astrocytic processes beyond the diffraction limit of conventional EM. In addition, the astrocyte morphology is more complex (especially in the human brain) that, one astrocyte is estimated to monitor an average range of 20,000–160,000 or 270,000–2 million individual synapses in rodent and human brain, respectively (Verkhratsky and Nedergaard, 2018). Moreover, astrocyte processes reach toward blood vessels (mostly capillaries) and assist in building up the BBB by enclosing capillary walls using their process terminals termed end-feet. This structurally arrangement helps them to regulate both synaptic activities and blood flow in the brain. Taken together, the ubiquitous distribution, complex morphologies, physical bridging synapses and blood vessels, expression and releasing of diverse signaling molecules, and strategical presence in the pathways of neuronal migration underlie astrocyte's myriad functions in the CNS including the OB (Figure 6).

Additionally, astrocytes in the OB not only form networks of processes that surround synapses, blood vessels and peri-vascular macrophages for stability (Sofroniew and Vinters, 2010; Ponath et al., 2018; Lawal et al., 2022) but also function in providing structural support to migrating neurons and in shaping connections between newborn neurons and existing neural circuits (Piet et al., 2004; Verkhratsky and Nedergaard, 2014).

#### Regulation of neuronal excitability and synaptic transmission

A series of synaptic-relevant proteins including Na/K ATPase (NKA), glutamate transporters (GLAST and GLT1), GABA transporters, GS and lactate transporters are expressed in astrocytic processes, the architectural elements of tripartite synapses. This enables astrocytes to regulate neuronal excitability and synaptic transmission via a number of mechanisms (Genda et al., 2011; Verkhratsky and Nedergaard, 2018). For instance, NKA aids astrocytes to uptake extracellular K^+^ at a concentration level resulting from action potential firing and neurotransmitter-gated ion channel opening; this is against the concentration gradient hence returning neuronal membrane potential to resting state and regulating neuronal excitability (McGrail et al., 1991; Bellot-Saez et al., 2017; Melone et al., 2019). The other key astrocytic role in regulating neuronal excitability is that the GLAST and GLT1 on astrocytes scavenge high amounts of the excitatory transmitter glutamate from the extracellular space enabling later release in accordance with requirements (Schousboe, 2003; Rose et al., 2020). This prevents the accumulation and consequently potential toxic effects of glutamate. Moreover, astrocytic GS converts glutamate into glutamine that is then released into extracellular space and taken up by neurons as a substrate for synthesis of the major inhibitory neurotransmitter GABA (McKenna et al., 2016). Additionally, astrocytes express the connexin 43 (Cx43) protein that creates channels across cells via gap junctions allowing important homeostatic functions such as neurotransmitter buffering (Nagy et al., 1999; Roux et al., 2011). Finally, astrocytes uptake lactate through specific lactate transporters and use it as an energy substrate for supporting synaptic transmission and plasticity. Besides these homeostatic functions, astrocytes also secrete gliotransmitters such as glutamate, GABA or ATP to proceed, via specific receptors in pre- and post-synaptic neuronal units, regulation of synaptic transmission and plastic changes (Theodosis et al., 2008; Harada et al., 2016; Covelo and Araque, 2018; Kofuji and Araque, 2021; Murat and García-Cáceres, 2021).

Although the aforementioned roles of astrocytes in controlling neuronal excitability and synaptic transmission/plasticity supported by increasing experimental evidence in other brain areas, we speculate similar astrocytic functions to be generally applicable in the OB.

Intriguingly, OB glomerular astrocytes are organized in such a unique way that their cell bodies surround each glomerulus while their processes are oriented toward the glomerulus center (Bailey and Shipley, 1993; De Saint Jan and Westbrook, 2005), where neuronal transmission is most intense (Roux et al., 2011). This strategic orientation is speculated to enhance communication within individual glomeruli while reducing cross-communication with nearby glomeruli. Indeed, olfactory nerve-evoked potassium currents and glutamate transporter currents were recorded in glomerular astrocytes with patch clamp recording in slice preparations, consistent with the predominant distribution of the inwardly rectifying K+ channel subunits Kir4.1/Kir5.1 and high level of glutamate transporters in glomerular astrocytes (Higashi et al., 2001; Utsumi et al., 2001; Hibino et al., 2004). The astrocytic potassium current depends on AMPA and NMDA receptors in MTCs while the glutamate transporter current is induced by glutamate released from both the olfactory nerve terminals and MTC dendrites (De Saint Jan and Westbrook, 2005), demonstrating not only the active role of these glial cells in detecting sensory input and limiting spread of intraglomerular excitation to adjacent glomeruli but also active astrocyte-neuron interactions in the glomerular circuits. The glomerular astrocyte-neuron interaction has further supported by studies showing that the fluctuation of membrane potential in glomerular astrocytes correlates with local field potential (Roux et al., 2011), which reflects local neural network activities. This correlation implies that astroglial networks could significantly influence olfactory information processing, potentially affecting odor discrimination and perception. This speculation was supported by a recent study with bidirectional modulation of OB astrocytes with chemogenetic approaches in behaving mice showing that: (1) activation of the excitatory DREADD hM3Dq in OB astrocytes *in vivo* decreased neuronal Ca^2+^ responses to odor stimulation and improved animal's performance in an olfactory learning task; (2) stimulation of the inhibitory DREADD hM4Di in OB astrocytes caused an increase in local neuronal intracellular Ca^2+^ levels but resulted in less accurate odor detection performance (Ung et al., 2020). Another important function of glomerular astrocytes is to couple neuronal activities with blood flow (Petzold et al., 2008; Otsu et al., 2015). These findings provide mechanistic bases for previous work showing that odor stimulation in whole animals leads to hyperemia (Kida et al., 2002; Chaigneau et al., 2003, 2007).

Furthermore, astrocytes release GABA to initiate hyperpolarizing inhibitory currents on mitral cells (Kozlov et al., 2006). These currents manifest synchrony among adjacent neurons, transiently inhibit neuronal activity for a duration of several 100 milliseconds. Alongside, glutamate release from astrocytes activates granule cell NMDA receptors making it a unique modulation of the olfactory circuitry (Kozlov et al., 2006). How this glia-neuron interaction contributes to the behavioral outcome warrant future exploration.

### Microglia

Microglia execute a critical and diverse array of functions in the OB. One of such prominent roles is that they synergistically involve in neuro- and synaptogenesis by contribute to the development, maintenance, and plasticity of synapses onto adult-born neurons (Figure 6). This microglial function is most abundantly observed in the maturation stage of adult-born granule cells (abGCs). For example, depleting microglia through conditional ablation induces reduced numbers of dendritic spines and excitatory inputs onto abGCs (Wallace et al., 2020). Moreover, the control of total neuron count during various physiological and pathological conditions is predominantly regulated by CX3CR1 signaling, a line of communication linking microglia and neurons (Reshef et al., 2017).

In addition to their involvement in generation of functional mature neurons, OB microglia are experimentally indicated to contribute to establishing neuronal circuitry in the both healthy and injured OB by phagocytosing adult-born neurons and providing the same function to neonatal-born neuronal varicosities, particularly when the animals are deprived of their olfactory sense (Denizet et al., 2017). On the other hand, as the native immune cells, microglia are indispensable for combatting bacterial pathogens and preserving CNS integrity. Thus, they establish the CNS first-line-of-defense to inhibit pathogen entrance (Herbert et al., 2012). Besides the cardinal function in innate immunity, microglia in the OB can switch from quiescent to vigorous phagocytosis to mold and nurture the brain parenchyma (Var and Byrd-Jacobs, 2019).

### OLs

OLs in the CNS primarily function to produce myelin, an insulating sheath that promotes expeditious electrical signal conduction in axons, augmenting neural communication and neuron survival (Bunge, 1968) (Figure 6). Inherent to this fundamental process are their multifaceted functions including the ion and water homeostasis, metabolic support for the neurons and synaptic functions, preserving the integrity of axons, regulating the thickness of axons, and modulating the clustering of sodium channels at the axonal node of Ranvier during axogenesis (Stadelmann et al., 2019). Retrospective analysis has identified a myriad of OL subtypes that have adaptive and plastic features equipping them to respond to the alterations in the CNS like the complex motor learning. Notably, several pieces of evidence imply that OLs have certain impact on the neuronal activities, which may involve the release of some signaling molecules, or direct physical contacts between OLs and neurons (Yamazaki, 2019). To maintain or favor the neuronal wellbeing, they also provide the nutrients and remove the metabolic waste products (Philips and Rothstein, 2017). Intriguingly, a potential role for OLs in neuronal plasticity has been proposed for encompassing the nervous system's ability to adapt in response to new experiences (Pajevic et al., 2023). This adaptability could have broad implications for learning and memory (Munyeshyaka and Fields, 2022), potentially influencing the learning of new odors in the OB. The spatial arrangement of OLs within the hedgehog OB is non-uniform and their density is much greater in the outer layers, especially the glomerular and external plexiform layers, where many axonal connections require myelination (Valverde and Lopez-Mascaraque, 1991). Evidence also shows that OLs are prone to oxidative stress and excitotoxic factors due to their involvement in the energy-intensive myelin generation process (Pfeiffer et al., 1993; Ibarretxe et al., 2006; Czopka and Lyons, 2013). Their dysfunction or loss can lead to demyelination, or damage to the myelin sheath, a feature common in several neurodegenerative and demyelinating disorders (Peferoen et al., 2014). These conditions can result from autoimmune attacks, injuries, strokes, toxic insults, or genetic defects disrupting myelin production (Patro et al., 2022). Following OL loss, a regenerative process, remyelination, typically initiates through the differentiation of OPCs, the most proliferative cells in the CNS, into myelinating OLs to restore the myelin sheath. However, the efficiency of this process often diminishes in demyelinating disorders, highlighting the critical need for research into strategies for enhancing remyelination as a key aspect of human health. Though OLs have been painstakingly investigated within the CNS, their role in the OB, a region teeming with such glia, stands as an uncharted frontier. Unpredictable findings concerning sensory processing and neural health maintenance may one day emerge from further probing in this niche. Groundbreaking revelations from deciphering what OLs contribute to olfaction could shatter our present understanding of the neural basis of sensory perception, and expose their implication in the neurodegenerative disorders.

### OECs

#### Ensheathing OSN axons

**Figure 7:**
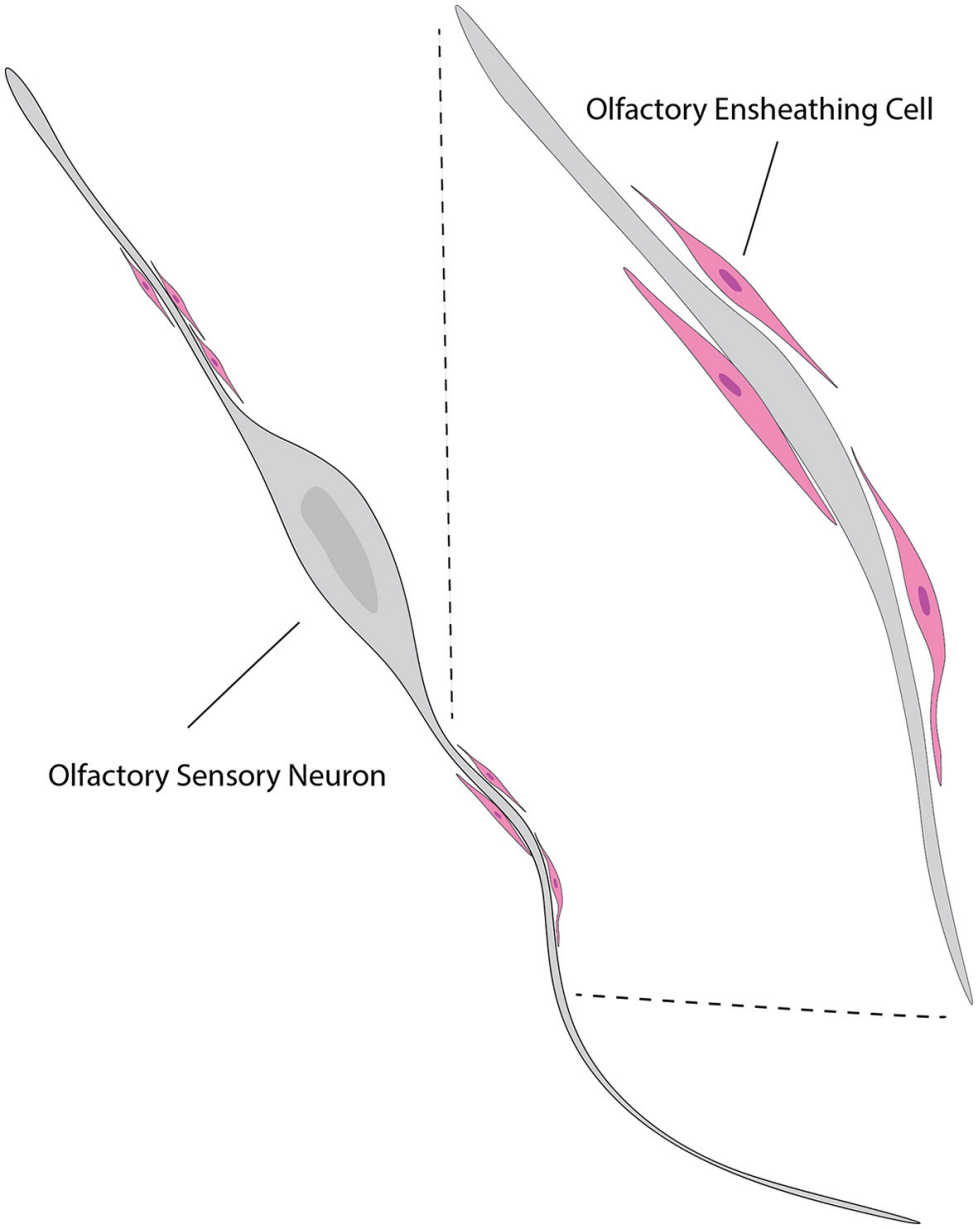
Illustration of the relationship between OECs and neurons. OECs (pink) are shown enveloping and supporting the axons of olfactory sensory neurons (gray). The close association between OECs and neuronal axons highlights their role in promoting axonal growth and guidance. This interaction is crucial for the continuous regeneration and proper functioning of the olfactory system. The inset shows a magnified view of the OECs wrapping around the axons, providing a supportive environment for axonal navigation and regeneration. This figure is referred from Windus et al. (2007).

In parallel to ensheathing the OSN axons as their main function, OECs are believed to be instrumental in directing regeneration of OSN axons from the olfactory epithelium to the OB both during regular cellular turnover and following injury (Higginson and Barnett, 2011) (Figure 7). Interestingly, the quantity of axons tends to decrease in the development of other peripheral nerves (Fraher, 1982) while the maturation of the olfactory nerves is characterized by a significant increase in the number of axons that are surrounded by OECs (Fraher, 1982), indicating the importance of OECs to functional OSNs.

Other functions of OECs include active participation in the intricate processes of olfactory nerve sorting, fasciculation, defasciculation, potentially contributing to the proper development and function of the olfactory system. For example, OECs in the inner ONL of the ventral OB produce varied guidance cues, including chemorepellant Sema3A in the cell membrane, a molecule critical for the proper ONL sorting of olfactory axons (Schwarting et al., 2000). Mice lacking Sema3A exhibit lowered ability to detect odors, indicative of their olfactory axons not being properly segregated in the ONL. These experimental findings demonstrate the critical roles of OECs and their respective signaling molecules in olfactory development. Although their involvement in these complex processes highlights the critical role of OECs in the olfactory system, further research is needed for fully understanding the underlying mechanisms by taking advantage of newly developed experimental approaches to manipulate OEC functionalities in these biological events.

#### Facilitating neuroregeneration

OEC's facilitation of neuroregeneration is evidenced by their secretion of numerous growth factors, such as NGF, basic fibroblast growth factors (bFBF), BDNF, glial cell line-derived neurotrophic factor (GDNF), CNTF, neurotrophins NT4, NT5, and neuregulins (Boruch et al., 2001; Woodhall et al., 2001; Lipson et al., 2003; Pellitteri et al., 2010; Russo et al., 2021; Ursavas et al., 2021; Stepanova et al., 2022). All these factors contribute to establishing an optimal milieu for neuronal growth, development and survival, indicating the possibility of using OECs for the treatment of neurodegenerative diseases and injuries in the CNS. In addition to the production of cell adhesion molecules and the secretion of growth factors, OECs have also been demonstrated to provide support for neuron survival and stimulate neurite outgrowth by their phagocytic potential. This biological process leads to more neurons with longer neurites and more elaborate branching patterns (Lipson et al., 2003). Another evidence of OEC roles in neurogenesis is that they facilitate the radial migration of the RMS by efficiently attracting neural progenitors (Zhu et al., 2010).

#### Therapeutic significance

OECs have emerged as a promising topic within the realm of cell-based therapies, especially for the treatment of spinal cord injury (SCI) models (Nandoe Tewarie et al., 2009). This translational effort has been not only based on their neuroprotective properties but also supported by findings that OECs promote axon regeneration and remyelination, leading to significant improvements in sensory and motor functions (Imaizumi et al., 1998).

### RGCs

#### Facilitation of neuroblast migration

The differentiation of RGCs into astrocytes is a key developmental event, indicative of the cellular plasticity and adaptability of RGCs in response to developmental requirements. Once transformed into astrocytes, these cells undertake the construction of the RMS, a defined migratory pathway that facilitates the neuroblasts' journey from the SVZ to the OB (Alvarez-Buylla et al., 2001) (Figure 1).

Although RGCs themselves do not directly participate in the migration process, their transformation into astrocytes represents an indirect but pivotal role in guiding neuroblast migration. Through differentiation into astrocytes, RGCs not only generate the key components of the RMS but also initiate the cascade of events that lead to successful neuroblast navigation to the OB (Ming and Song, 2011). This relationship between RGCs and astrocytes underscores the intricate and meticulously orchestrated mechanisms underlying neuroblast migration within the RMS, thus providing deeper insights into the complexities of brain development.

#### Phagocytosis of axonal debris

RGCs in the OB are implicated in the task of axonal debris clearance (Amaya et al., 2015). In a controlled study using Sox10-deficient mice, it was observed that the phagocytosis of olfactory axons debris by RGCs did not take place in the absence of Sox10 expression, which led to evidently defective development of the OB. The accumulation of olfactory axonal debris in mice without Sox10 throughout the depth of the bulb demonstrates the significant role of RGCs in axon overextension removal, especially in deeper part of OB. Thus, RGCs plays an extremely important role in maintaining the structural integrity via cleanliness of axonal debris within the OB tissue.

#### Organization of the OB layers

The distinctive spatial organization pattern of RGCs enables them to participate in the development of OB structural lamination. The cell bodies of these glia are positioned near the ventricle while their processes extend toward the periphery of the OB (Gonzalez and Silver, 1994).

A preference for preserving olfactory axons seems to localize RGCs to the outer layers of the OB, which is coincident with the localization of their processes. In contrast, degrading axon fragments are found mostly deeper within the bulb, near the ventricle where RGC bodies sit. This orientation of the axons implicates roles of RGCs in the proper positioning of axons within the OB. Thus, the orchestrated spatial distribution of RGCs may serve to influence axonal pathfinding and the final architecture of the OB layers.

## Interactive relationship

### RGCs vs. astrocytes

Prominently established in the SVZ, RGCs act as central progenitors throughout gliogenesis and neurogenesis in the embryonic brain (Alvarez-Buylla et al., 2001; Doetsch, 2003). RGCs play an important role in these processes by constantly dividing to produce new neuroblasts. These newborn neurons, however, need direction and a hospitable environment to survive the brain's labyrinthine ways (Kriegstein and Alvarez-Buylla, 2009). The transformation of RGCs into astrocytes helps the system meet these requirements.

In the course of neurogenesis, RGCs divide asymmetrically and give life to two separate cell types: (1) RGCs; and (2) neurons, OLs and astrocytes (Alvarez-Buylla et al., 2001; Merkle et al., 2004; Menn et al., 2006). Neuronal progeny along the RGC fiber migrates to their terminal sites in the brain, such as the OB.

As neurodevelopment advances, the shift from neurogenesis to gliogenesis is a crucial juncture. It is during this time that RGCs undergo their transition into astrocytes (Merkle et al., 2004; Ventura and Goldman, 2007). RGC morphological alterations characterize this process, i.e., radial processes retract and the cell body enlarges. Moreover, a change in gene expression profiles corresponds to this transformation. For instance, astrocyte-specific genes including the GFAP become significantly upregulated in these emerging astrocytes (Eng, 1985).

At the mechanistic level, intricate angiogenic pathways and extracellular signals are found to underlie the elaborate process of cellular differentiation. Upon activation of RGCs, notch as a key intracellular mediator induces the expression of glial genes in RGCs and drives them to an astrocytic fate (Gaiano and Fishell, 2002). Furthermore, the astrocytic differentiation from RGCs is promoted by signals from bone morphogenetic proteins (BMPs), the transforming TGFβ superfamily and the JAK-STAT pathway (Bonni et al., 1997). Moreover, the extracellular environment also contributes significantly to the RGC differentiation process. For example, cell-cell interactions as well as the presence of growth factors and cytokines within the extracellular space exert a substantial influence over the RGC's cell fate decisions.

Once the differentiation process within the SVZ wraps up, the produced astrocytes migrate to their various intended destinations, including the OB (Gross et al., 1996) where they integrate into the preexisting neural circuitry to play vital supporting and neuromodulatory roles. Again, it is important to point out that this overview provides merely a simplified version of the intricate and multifaceted process by which RGCs differentiate specifically into astrocytes. Thus, ongoing investigations in neural stem cell biology promise to provide a more nuanced understanding of the relationship between these two subpopulations of glial cells.

### RG2Cs vs. OLs

In the OB, RGCs have been found to be progenitors of OLs in a study with a lentivirus-based labeling technique in mice with a floxed reporter suitable for long-term fate mapping (Anderson et al., 1997; Ventura and Goldman, 2007). The lentivirus was delivered to subpial and layer I astrocytes, corresponding to the sites of subpial endfeet of the dorsal radial glia. To accurately track the localization and subsequent fate of the labeled cells, analyses of the mice were carried out at a variety of time points, specifically 4 days, 3 weeks, and 8 weeks post-viral delivery. With this approach, researchers mapped dorsal radial glia in mice and revealed OPCs derived from dorsal RGCs within both the cortex and subcortical white matter (Staugaitis et al., 2001). Identified through the expression of the OL precursor marker NG2, these OPCs were a testament to the origins of RGCs.

The capacity of dorsal RGCs to become OPCs and OLs in the postnatal OB has been supported by growing evidence (Anderson et al., 1997; Staugaitis et al., 2001; Ventura and Goldman, 2007), highlighting the exceptional malleability of RGCs and their pivotal role in promoting neuronal activity through the process of myelination.

### OECs vs. astrocytes

OECs have been observed to freely intermingle with astrocytes in co-cultures (Lakatos et al., 2000). This could be an important factor during early olfactory system development when olfactory axons are sending outgrowth cones to make synaptic connections with the primary dendrites of mitral cells. Research has also shown that when reactive astrocytes are co-cultured with OECs, these astrocytes exhibit significantly reduced GFAP immunoreactivity, suggesting that OECs not only inhibit proliferation of astrocytes but also putatively suppress hypertrophy and further underline the complex interaction of OECs with astrocytes or vice-versa (O'Toole et al., 2007).

A recent study centered on Ca^2+^ signaling within panglial networks disclosed the intercellular communication between OECs and astrocytes in regulating blood vessel sizes in the mouse OB using in-toto preparations. It turns out that Ca^2+^ transients in astrocytes in the OB GL trigger a delayed Ca^2+^ response in OECs in the ONL, a process reliant on gap junctional coupling. This astrocyte-to-OEC transmission leads to vasoconstriction of OEC-associated blood vessels in the ONL. The authors posit that this mechanism could be pivotal in regulating blood flow in the OB and potentially other brain regions (Beiersdorfer et al., 2019). This mechanism offers insights into neurovascular coupling and its role in neurological disorders such as stroke, AD, and epilepsy.

### RGCs vs. OECs

The intricate interactions between RGCs and OECs play a pivotal role in the meticulous development of the OB as both cell types contribute to the formation and maintenance of olfactory sensory neuron axons.

OECs are highly populated in the superficial nerve fiber layer but not in the deeper regions of the OB whereas RGC processes are mainly confined to the central region of the OB in mouse embryos (Amaya et al., 2015). However, in mice lack of the transcription factor Sox10, which is essential for CNS development, OECs neither proliferate nor migrate effectively leading to a significant reduction OECs in the OB nerve fiber layer (Barraud et al., 2013) whereas RGC processes extensively extend all the way into this superficial nerve fiber layer, indicating a repulsive cue produced by the OECs within this layer to repel RGC processes. These findings highlight the importance of interactions between OECs and RGCs to the development and the subsequent formation of the OB.

### Astrocytes vs. microglia

Astrocytes participate in the regulation of brain homeostasis through the activation of Type I interferon (IFN) pathway (Greter et al., 2012). These regulatory functions are not only important for keeping the neuronal environment stable, but also have a protective function. Indeed, astrocytes, by controlling microglial activity, are crucial for an efficient antiviral defense and hence brain function safeguard (Vasek et al., 2016). Maintaining homeostasis in the CNS requires the dynamic interplay between these two types of glial cells. Astrocytes have long been recognized for their role in maintaining the BBB integrity and for regulating synaptic transmission in the CNS and are now known to communicate bi-directionally with microglia (Tasdemir-Yilmaz and Freeman, 2014) (Figure 6), a process crucial to the neuronal network in the OB and being enhanced during stress such viral encephalitis (Detje et al., 2009). Astrocytic signaling influences a host of processes including synaptic pruning, and the response to neuroinflammation (Vainchtein et al., 2018).

Interestingly, type I IFN receptors are essential to the communication between astrocytes and microglia by regulating microglial activation (Müller et al., 1994). These receptors on astrocytes initiate the signaling pathway which has profound consequences on microglia, in particular in the instance of viral attack within the CNS (Daniels et al., 2017). This astrocyte-microglia connection exhibits dynamism as it adjusts to the physiological and pathological states in the CNS. Astrocytes are a direct contributor to the course of immune responses during viral infection and are not bystanders to simple contacts with the microglia (Prinz et al., 2008). For example, in the context of viral encephalitis, astrocytes respond strongly by using their interferon receptors to regulate the activation of the microglia and the signaling of interferon receptors, which is assumed to play a crucially regulatory role in determining the magnitude and kind of microglial activation (Kalinke et al., 2011; Nayak et al., 2013). The regulatory system of this glial interaction is responsible for maintaining a delicate equilibrium, allowing for an appropriate immune response to clear pathogens while also preventing harmful neuroinflammation (Pfefferkorn et al., 2016). Thus, the signaling pathways mediated by IFNs in astrocytes appear to be protective in limiting the extent of pathogenesis in neurotropic viral infections and a participant in maintaining the immunological privilege within the CNS (Hwang and Bergmann, 2018).

Microglia participate in the refinement of OB circuits, particularly in postnatal developmental stages (Tremblay et al., 2010). The precision of olfactory sensory maps is ensured through the optimization of synaptic connections and the elimination of surplus neurons and synaptic elements, achieved through mechanisms such as phagocytosis (Paolicelli et al., 2011). However, in neurologically pathogenic circumstances, the interplay between microglia and astrocytes becomes more pronounced wherein astrocytes secrete cytokines to stimulate microglia, hence producing a reactive state (Liddelow et al., 2017).

At the molecular level, the cross-talk between astrocytes and microglia is mediated by a number of signaling molecules and cytokines. For instance, astrocytes release cytokines including interleukin-33 to modulate microglial activity including synaptic engulfment as observed in the context of neuroinflammation. In turn, microglial cells secrete factors such as TGF-β to affect astrocyte functions (Butovsky et al., 2014; Vainchtein et al., 2018). However, both glial types respond to purinergic signaling and synergize in response to stressors such as hypoxia/neurodegenerative diseases (Lou et al., 2016). In condition like AD, dissociation of this interaction contributes to aggravation of the disease. These examples highlight the contribution of the astrocyte-microglia axis to neurodegenerative pathologies (Lian et al., 2016).

In addition, astrocytic and microglial interactions are also integral in the dynamic turnover of neurons within the OB. Neuroblasts must not only find their way from the SVZ to the OB properly, but also learn to integrate within existing brain circuitry. These biological processes are facilitated by the collective actions of these two types of glia (Whitman and Greer, 2009). Astrocytes are pivotal in the migration and differentiation of neuroblasts, while microglia positively impact the proliferation and differentiation of neuroblasts through contact-mediated signaling. Consequently, a close spatial and temporal dialogue between astrocytes and microglia is thought necessary for continuous regeneration of olfactory neurons (Whitman and Greer, 2009).

Collectively, astrocyte and microglia interaction in the OB play a major role in sustaining olfactory circuits structurally and functionally (Matejuk and Ransohoff, 2020). The interaction of these glial cells is extremely important in maintaining synaptic homeostasis as well as immune responses. Given their indispensable roles in the complex process of olfactory signaling, a comprehensive understanding of the underlying mechanisms will potentially pave the way to novel therapeutic approaches specifically tailored for olfactory diseases (Kettenmann et al., 2013).

### Astrocytes vs. OLs

In the adult CNS, OLs primarily arise from OPCs and are abundant in both white and gray matter (Figure 1). Interactions between astrocytes and OPCs are necessary for guiding OPC migration and adult OPC maturation into OLs. These complex glial interactions are not only important for normal CNS development but also for pathology or potential repair mechanisms of demyelinating diseases. OPCs originate from specific regions of the neural tube and migrate through CNS, an action considerably influenced by astrocytes (Figure 6). For example, astrocytes secrete numerous cues, such as semaphorins, Shh (Sonic Hedgehog) and FGF-2 (Fibroblast Growth Factor-2) to impact OPC behaviors (Clemente et al., 2011). These cues are important in either directly driving the response to important signals or aiding in the generation of signal gradients *in vivo* (Clemente et al., 2011).

Furthermore, astrocytes produce extracellular matrix for OPCs to attach and then crawl along. This is particularly evident during CNS development and repair as OPCs extend specialized processes and travel within white matter. Astrocytes also store PDGF, which is critical to the survival and proliferation of OPCs (Barres et al., 1993; Chernausek, 1993; Gard et al., 1995; Moore et al., 2011). This astrocyte-OPC relationship is interesting in the context of demyelinating diseases such as multiple sclerosis, in which both astrocytes and microglia profoundly impact OL biology (Ransohoff and Perry, 2009).

Although the focus here is on the CNS, similar or analogous interactions may occur in other parts of the nervous system such as the OB. On the other hand, since the OB is cytoarchitecturally and neurochemically different from other parts of the brain, the specifics of these glial interactions likely differ thus are certainly worth future research effort.

Taken together, the biological significance of glia-interations depends on their unique functional features but with ultimate goals to ensure proper neural development and maintain appropriate environments for neural circuitry and overall brain function. Specifically, astrocytes provide essential structural and metabolic support, regulate neuronal excitability, and facilitate synaptic transmission, while also participating in neurogenesis and responding to neurological disorders. Microglia contribute to neuro- and synaptogenesis, phagocytose adult-born neurons, and serve as the first line of defense against pathogens, maintaining CNS integrity. Oligodendrocytes are primarily responsible for myelination, which ensures rapid electrical signal conduction and supports neuronal survival, playing roles in ion and water homeostasis, and potentially influencing neuronal plasticity. OECs are crucial for guiding the regeneration of OSN axons and facilitating neuroregeneration through the secretion of growth factors. RGCs contribute to the migration of neuroblasts and phagocytosis of axonal debris, maintaining the structural integrity of the OB. The synergistic interplay between these glial cells, such as astrocytes and microglia in immune responses, astrocytes and OLs in guiding OPC migration, and OECs and RGCs in olfactory axon formation, highlights the complex and coordinated efforts required to maintain the OB structural and functional integrity, ultimately influencing sensory perception and response to neurological challenges.

## Involvement in neurological disorders

### Alzheimer's disease

Alzheimer's Disease (AD) involves selective damage to brain regions essential for cognition and memory, such as the neocortex, hippocampus, amygdala, basal forebrain cholinergic system, and brainstem monoaminergic nuclei (Rasool et al., 1986). Its hallmark pathological features include β-amyloid plaque deposition and hyperphosphorylated tau neurofibrillary tangles, resulting in neurotoxicity, synaptic loss, and neuronal degeneration (Bloom, 2014). In the AD pathogenic OB, astrocytes and microglia become reactive, releasing pro-inflammatory cytokines, chemokines, and reactive oxygen species, which exacerbate neuronal damage. These glial cells also play a role in clearing Aβ peptides; however, their dysfunction in AD impairs this process, resulting in Aβ accumulation and plaque formation. Astrocytes secrete neurotrophic factors like NGF and BDNF, which support neuronal survival, and their reduced secretion in AD further contributes to neuronal degeneration and cognitive deficits (Doorn et al., 2014; Marei et al., 2015). Microglia exhibit increased density in the AON of AD patients, associated with deposits of β-amyloid, hyperphosphorylated tau, and α-synuclein (Doorn et al., 2014; Carmona-Abellan et al., 2021). The insulin-degrading enzyme (IDE), a molecule promoting the degradation of amyloid-β in the brain and improving cognitive impairment in AD patients, modulates microglial activity. Consistently, IDE knockout (IDE-KO) mice show region-specific microgliosis and variations in myelin phagocytosis, which affect cytokine production and stress responses (Corraliza-Gomez et al., 2023). These microglia can be either neuroprotective or neurotoxic through their production of reactive oxygen species and cytokines. SAK3 has demonstrated efficacy in reducing oxidative stress and cognitive impairments in an AD mouse model by enhancing T-type calcium channel activity and stimulating α7 nicotinic acetylcholine receptors, thereby promoting a neuroprotective phenotype (Yuan et al., 2021). OLs, which are responsible for myelination in the CNS, are compromised in AD, leading to disrupted neural transmission in the OB and contributing to early olfactory deficits. Targeting OLs for myelin repair and neuroprotection could alleviate these symptoms (Franco et al., 2024). Additionally, OECs show promise in treating AD by promoting axonal regeneration and enhancing amyloid-beta clearance. Their neuroprotective and anti-inflammatory properties help mitigate neuroinflammation and neuronal damage, supporting synaptic plasticity and counteracting dysfunction observed in AD (McLaren and Kawaja, 2024).

### Parkinson's disease

Parkinson's Disease (PD) is the second most common neurodegenerative disorder after AD, characterized by the selective loss of dopaminergic neurons in the substantia nigra pars compacta and the presence of Lewy bodies. This dopaminergic deficiency leads to motor symptoms such as tremor, rigidity, and bradykinesia, as well as non-motor symptoms including olfactory deficits as well as cognitive and psychiatric disturbances (Rodriguez-Oroz et al., 2009). Astrocytes contribute to the neuroinflammatory environment in PD through the release of IL-1β via the JAK2-STAT3 signaling pathway, exacerbating neuronal damage (Yang et al., 2019). Similarly, microglial activation, observed in chronic nasal inflammation, upregulates pathways like TLR4, MyD88, and NF-κB, which are linked to neurodegeneration (Lu et al., 2021). These activated microglia further contribute to neuroinflammation, evidenced by increased CD68 immunoreactivity and IL-1 family gene expression (Vroon et al., 2007). Inhibiting these pathways has shown potential in mitigating inflammation, thus slowing down PD progression (Wang et al., 2023). OLs, essential for myelination in the CNS, are compromised in PD, leading to disrupted neural signaling. The accumulation of α-synuclein aggregates impairs OL function, worsens myelin degradation and neuronal dysfunction (Murray et al., 2022). Thus, enhancing OL function and promoting myelin repair are promising therapeutic strategies for PD. Additionally, OECs have been shown to improve the survival of dopaminergic neurons in PD models by enhancing motor function recovery and reducing immune responses at the graft site. This co-grafting strategy leads to better outcomes in PD models, highlighting the therapeutic potential of OECs (Weng et al., 2020).

### Amyotrophic lateral sclerosis

Amyotrophic lateral sclerosis (ALS) is a rapidly progressive and fatal neurodegenerative disease characterized by the degeneration of motor neurons in the brain, brainstem, and spinal cord, leading to muscle weakness and eventual respiratory failure. The pathological features include intracellular inclusions and motor neuron loss, with both genetic and sporadic forms contributing to the disease's complexity (Goutman et al., 2022). In the intricate pathophysiology of ALS, astrocytes and microglia emerge as central players orchestrating a complex interplay between neuroinflammation and neurodegeneration. Astrocytes undergo a transformative process of reactive gliosis, unleashing a cascade of inflammatory mediators that perpetuate neuronal injury, while activated microglia unleash a storm of pro-inflammatory cytokines, fueling a vicious cycle of neuroinflammation and neurotoxicity (Ringer et al., 2017). OLs in the OB may have implications for the treatment of ALS by potentially influencing myelination processes and providing support to motor neurons (Martin and Liu, 2007). OECs have demonstrated the ability to enhance the survival of both upper and lower motor neurons in ALS by promoting regeneration and remyelination of damaged spinal pathways. This therapeutic approach holds promise for improving disease progression and neurological function recovery in ALS patients (Li et al., 2013).

### Multiple sclerosis

Multiple sclerosis (MS) is an autoimmune disease that targets the CNS, where the immune system mistakenly attacks the myelin sheath protecting nerve fibers, leading to disrupted nerve signal transmission. This results in a range of symptoms, including vision problems, muscle weakness, and coordination issues, with disease progression varying between relapsing and progressive forms (Dobson and Giovannoni, 2019). In MS, activated astrocytes exacerbate neuroinflammation and tissue damage by secreting pro-inflammatory mediators. Similarly, microglia become activated in response to inflammatory stimuli, releasing pro-inflammatory cytokines and reactive oxygen species, which perpetuate neuroinflammation and neuronal injury (DeLuca et al., 2015). Elevated levels of TNFα in MS patients contribute to oligodendrocyte apoptosis and demyelination, worsening disease pathology and hindering remyelination. Prolonged TNFα exposure has been shown to cause diffuse oligodendrocyte death and chronic CNS demyelination in transgenic mice, mirroring the MS phenotype. Unlike Schwann cells, olfactory OECs thrive under high TNFα levels due to their ability to downregulate TNFα receptors. This unique capability positions OECs as promising candidates for promoting remyelination and neural repair, potentially restoring myelin and improving neural function in MS patients (Lankford et al., 2017).

### Huntington's disease

Huntington's Disease (HD), characterized by striatal and cortical degeneration due to CAG repeat expansions in the Huntington gene, leads to severe neuronal loss and ultimately death with olfactory deficit as one of the common symptoms (Barnat et al., 2020). In HD, astrocytes derived from induced pluripotent stem cells (iPSCs) of patients exhibit abnormal characteristics such as cytoplasmic vacuolation, contributing to neuronal dysfunction and degeneration. Dysfunctional astrocytes in the OB exacerbate HD pathology by impairing neuronal support, making iPSC-derived astrocytes valuable for modeling HD and identifying therapeutic targets (Juopperi et al., 2012). Microglial activation in the OB leads to neuroinflammation and neuronal dysfunction, as they proliferate around mutant huntingtin-expressing neurons. Mesenchymal stem cell (MSC) therapy shows potential in modulating microglial activity, reducing inflammation, and promoting neuroprotection in HD. Targeting microglial function in the OB through MSC-based interventions could mitigate neuroinflammatory processes and support neuronal health (Yu-Taeger et al., 2019). The dysfunction of OLs in the OB significantly impairs neural signal transmission and exacerbates neurodegeneration in HD. Enhancing the health and function of these glial cells offers a promising therapeutic strategy. Genetically modified neural stem/progenitor cells that differentiate into OLs could restore myelin integrity and improve neural function (Marei et al., 2013). Although research on OECs in HD treatment is limited, studies suggest that transplanting active cells can provide therapeutic benefits such as neurotrophic factor secretion, reduction of neuroinflammation, enhancement of neuronal plasticity, and cell substitution (De Gioia et al., 2020). While further research is needed to confirm the efficacy of OEC transplantation in HD, the potential for this cell therapy as a treatment strategy remains promising.

### Lewy body dementia

Lewy body disease (LBD) is a prevalent neurodegenerative disorder characterized by the accumulation of alpha-synuclein protein deposits, known as Lewy bodies, in the brain. This disease manifests through a range of symptoms, including cognitive impairment, movement disorders, and autonomic dysfunction. LBD often co-occurs with AD and PD, complicating diagnosis and treatment (Samudra et al., 2024). In patients with LBD, the density of microglia and astrocytes in the OB is significantly increased. The activation of microglia is associated with a neuroinflammatory response, which is a key feature of LBD pathology. Similarly, the increase in astrocytes reflects an inflammatory environment that may exacerbate neuronal damage in the disease (Carmona-Abellan et al., 2021). OLs in LBD, disrupted by α-synuclein accumulation, impair myelination and contribute to neural degeneration, particularly in the OB, leading to sensory deficits like the loss of smell in PD. Therapeutically, targeting oligodendrocytes to mitigate α-synuclein toxicity, enhancing myelin repair, and reducing neuroinflammation offer a promising approach to supporting neuronal health (Nakamura et al., 2016).

### Neurodevelopmental disorders

Neurodevelopmental disorders constitute a heterogeneous group of conditions originating from disruptions in brain development, resulting in a spectrum of cognitive, motor, and behavioral impairments. These disorders typically manifest in early childhood and frequently persist into adulthood, significantly impacting an individual's functional capabilities and quality of life (Thapar et al., 2017). Disruptions in RGC function can lead to a range of neurodevelopmental disorders, particularly affecting the olfactory system and other CNS regions. These disruptions result in aberrant neuronal migration, leading to conditions such as Kallmann syndrome, characterized by anosmia and hypogonadotropic hypogonadism (Cariboni and Maggi, 2006; Amaya et al., 2015). RGC dysfunction also impacts interneuron migration in the OB and neocortex, causing lissencephaly and micro lissencephaly, which result in severe developmental delays and neurological issues (Wu and Wang, 2012). Mutations in genes such as Lis1 and Nde1, crucial for RGC function, are linked to these disorders (Reiner et al., 1993; Alkuraya et al., 2011). Additionally, RGCs are involved in cerebral cortical vascularization, and their dysfunction can lead to neonatal cerebral hemorrhage and gliomas, common and often lethal brain tumors (Vick et al., 1977; Ma et al., 2013). Understanding the mechanisms of RGCs actions is essential for addressing these severe neurodevelopmental disorders and improving treatment strategies.

## Conclusions

In this review, we provide a comprehensive overview of the origination, migration, morphology, biomarkers, and function as well as interactive relationship of glia cells in the mammalian OB with the aim to identify what is known or yet to know thus providing an informative reference point for future research effort especially for the olfactory neuroscience field. Additionally, we emphasize the indispensable roles of glial cells in the formation and function of the OB as well as their involvement in neurodegenerative diseases such as AD, PD, and MS.

Although significant progress on the general biology of individual glial cell types in the mammalian OB has been achieved since their initial recognitions or identification, there are many open questions worth future research effort as pointed out in the prior individual sections. Particularly, given the advent of many cutting-edge technologies like chemogenetics or cell type specific *in vivo* imaging to manipulate or monitor glial activities, investigation of the functional glia-glia or glia-neuron interactions and their contribution to neuronal and network operations in olfactory system will not only significantly advance our understanding of the neurobiological mechanisms of signal processing in health but also provide physiological insights into developing potential approaches or strategies for early and accurate diagnosis of different neurological disorders. Moreover, the non-invasive imaging approaches including functional magnetic resonance imaging or spectroscopy and positron emission tomography enable assessing OB glial cell activities or pathological or biochemical consequences in the brain of patients with neurological or olfactory disorders. Cell type specific genomics, proteomics, single cell profiling or RNA sequencing will facilitate to bring the mechanistic analysis of the functional or dysfunctional outcomes to the gene, transcriptional, protein or intracellular signaling levels.

Another future research area could be to include glial cells in disease modeling, drug and biomarker discovery and therapeutic testing as it is important to study glial cell behaviors in different relevant diseases and their responses to different treatments.

Given the increasing recognition of the olfactory deficit as a preclinical biomarker of multiple neurodegenerative disorders, we believe that future work focusing on the OB glial cells will open new avenues for early diagnosis and innovative treatments, ultimately improving outcomes for individuals with neurodegenerative diseases.

## Author contributions

DZ: Conceptualization, Investigation, Validation, Writing – original draft. MH: Conceptualization, Investigation, Validation, Writing – original draft. SL: Conceptualization, Funding acquisition, Project administration, Resources, Supervision, Writing – review & editing.

## Figures and Tables

**Table A1:** Glossary of professional terms.

AEP	Anterior entopeduncular area	MS	Multiple sclerosis
ALS	Amyotrophic lateral sclerosis	NSCs	neural stem cells
AD	Alzheimer's Disease	NKA	Na/K ATPase
aSVZ	Anterior subventricular zone	OB	olfactory bulb
abGCs	Adult-born granule cells	OSNs	Olfactory sensory neurons
BBB	Blood-brain barrier	OLs	Oligodendrocytes
BMPs	Bone morphogenetic proteins	OECs	Olfactory ensheathing cells
bFGF	Basic fibroblast growth factor	OPCs	Oligodendrocyte progenitor cells
CNS	Central nervous system	OBNSCs	OB neural stem cells
DCs	Dendritic cells	ONECs	Olfactory nerve ensheathing cells
EPL	External plexiform layer	OEG	Olfactory ensheathing glia
EGFR	Epidermal growth factor receptor	ONL	Olfactory nerve layer
FGFR	fibroblast growth factor receptor	PD	Parkinson's Disease
GL	Glomerular layer	RGCs	Radial glia cells
GCL	Granule cell layer	RMS	Rostral migratory stream
HD	Huntington's Disease	RE	Rostral extension
IPL	Internal plexiform layer	RC2	RGC cell marker 2
IFN	Type I interferon	SVZ	Subventricular zone
IDE	Insulin-degrading enzyme	SCWM	Subcortical white matter
IDE-KO	IDE knockout	VEGF	Vascular endothelial growth factor
LBD	Lewy body disease	V-SVZ	Ventricular-SVZ
MTCs	Mitral and tufted cells	WM	White matter
MCL	Mitral cell layer	YS	Yolk sac
MGE	Medial ganglionic eminence		
